# New Insights in the Era of Clinical Biomarkers as Potential Predictors of Systemic Therapy-Induced Cardiotoxicity in Women with Breast Cancer: A Systematic Review

**DOI:** 10.3390/cancers15133290

**Published:** 2023-06-22

**Authors:** Alexia Alexandraki, Elisavet Papageorgiou, Marina Zacharia, Kalliopi Keramida, Andri Papakonstantinou, Carlo M. Cipolla, Dorothea Tsekoura, Katerina Naka, Ketti Mazzocco, Davide Mauri, Manolis Tsiknakis, Georgios C. Manikis, Kostas Marias, Yiola Marcou, Eleni Kakouri, Ifigenia Konstantinou, Maria Daniel, Myria Galazi, Effrosyni Kampouroglou, Domen Ribnikar, Cameron Brown, Georgia Karanasiou, Athos Antoniades, Dimitrios Fotiadis, Gerasimos Filippatos, Anastasia Constantinidou

**Affiliations:** 1 A.G. Leventis Clinical Trials Unit, Bank of Cyprus Oncology Centre, 32 Acropoleos Avenue, Nicosia 2006, Cyprus; elisavet.papageorgiou@bococ.org.cy (E.P.); marina.zacharia@bococ.org.cy (M.Z.); 2 2nd Department of Cardiology, Attikon University Hospital, National and Kapodistrian University of Athens, 12462 Athens, Greece; keramidakalliopi@hotmail.com; 3 Cardiology Department, General Anti-Cancer Oncological Hospital, Agios Savvas, 11522 Athens, Greece; 4 Department of Oncology-Pathology, Karolinska Institute, 17176 Stockholm, Sweden; andri.papakonstantinou@ki.se; 5 Department for Breast, Endocrine Tumours and Sarcoma, Karolinska University Hospital, 17176 Stockholm, Sweden; 6 Cardioncology and Second Opinion Division, European Institute of Oncology (IEO), IRCCS, Via Ripamonti 435, 20141 Milan, Italy; carlo.cipolla@ieo.it; 7 2nd Department of Surgery, Aretaieio University Hospital, National and Kapodistrian University of Athens, 76 Vas. Sofias Av., 11528 Athens, Greece; dtsekoura@med.uoa.gr (D.T.); effkamp@uoa.gr (E.K.); 8 2nd Cardiology Department, University of Ioannina Medical School, 45110 Ioannina, Greece; anaka@uoi.gr; 9 Applied Research Division for Cognitive and Psychological Science, European Institute of Oncology IRCCS, 20139 Milan, Italy; ketti.mazzocco@ieo.it; 10 Department of Oncology and Hemato-Oncology, University of Milan, 20122 Milan, Italy; 11 Department of Medical Oncology, University of Ioannina, 45110 Ioannina, Greece; dmauri@uoi.gr; 12 Department of Electrical and Computer Engineering, Hellenic Mediterranean University, 71410 Heraklion, Greece; tsiknaki@ics.forth.gr (M.T.); kmarias@ics.forth.gr (K.M.); 13 Computational BioMedicine Laboratory (CBML), Institute of Computer Science, Foundation for Research and Technology Hellas (FORTH), 70013 Heraklion, Greece; gmanikis@ics.forth.gr; 14 Department of Medical Oncology, Bank of Cyprus Oncology Centre, 32 Acropoleos Avenue, Nicosia 2006, Cyprus; yiola.marcou@bococ.org.cy (Y.M.); eleni.kakouri@bococ.org.cy (E.K.); ifigenia.konstantinou@bococ.org.cy (I.K.); myria.galazi@bococ.org.cy (M.G.); 15 Department of Radiation Oncology, Bank of Cyprus Oncology Centre, 32 Acropoleos Avenue, Nicosia 2006, Cyprus; maria.daniel@bococ.org.cy; 16 Division of Medical Oncology, Institute of Oncology Ljubljana, Faculty of Medicine, University of Ljubljana, Zaloska Cesta 2, 1000 Ljubljana, Slovenia; dribnikar@onko-i.si; 17 Translational Medicine, Stremble Ventures Ltd., 59 Christaki Kranou, Limassol 4042, Cyprus; cameron.brown@stremble.com; 18 Biomedical Research Institute, Foundation for Research and Technology, Hellas, 45500 Ioannina, Greece; g.karanasiou@gmail.com; 19 Research and Development, Stremble Ventures Ltd., 59 Christaki Kranou, Limassol 4042, Cyprus; athos.antoniades@stremble.com; 20 Unit of Medical Technology and Intelligent Information Systems, Department of Materials Science and Engineering, University of Ioannina, 45110 Ioannina, Greece; fotiadis@uoi.gr; 21 Cardio-Oncology Clinic, Heart Failure Unit, Department of Cardiology, National and Kapodistrian University of Athens Medical School, Athens University Hospital Attikon, 11527 Athens, Greece; gfilippatos@med.uoa.gr; 22 School of Medicine, University of Cyprus, Panepistimiou 1, Aglantzia, Nicosia 2408, Cyprus

**Keywords:** chemotherapy-induced cardiotoxicity, cancer therapy-induced cardiac dysfunction, clinical biomarkers, breast cancer, LVEF, BNP, troponins

## Abstract

**Simple Summary:**

Cancer therapy-related cardiac dysfunction (CTRCD) has been an urgent medical issue in patients that receive breast cancer therapies including anthracycline-based chemotherapies and/or targeted anti-HER2 therapies such as trastuzumab. Traditional biomarkers used as standard of care may be useful indicators of cardiac damage but their use to predict the onset of CTRCD lacks reliability. Ongoing clinical studies aim to explore new insights into the use of traditional biomarkers and investigate the promising role of novel biomarkers as reliable indicators and/or predictors of CTRCD. Patients with breast cancer could benefit from an alternative cardiac risk stratification plan that has the potential to predict the onset of CTRCD and/or detect CTRCD at early stages. The aim of this systematic review is to provide an overview of the human studies, which explore novel insights into traditional biomarkers and/or novel biomarkers that can be used for the early detection and/or prediction of CTRCD in breast cancer patients undergoing cardiotoxic-cancer therapies.

**Abstract:**

Cardiotoxicity induced by breast cancer therapies is a potentially serious complication associated with the use of various breast cancer therapies. Prediction and better management of cardiotoxicity in patients receiving chemotherapy is of critical importance. However, the management of cancer therapy-related cardiac dysfunction (CTRCD) lacks clinical evidence and is based on limited clinical studies. Aim: To provide an overview of existing and potentially novel biomarkers that possess a promising predictive value for the early and late onset of CTRCD in the clinical setting. Methods: A systematic review of published studies searching for promising biomarkers for the prediction of CTRCD in patients with breast cancer was undertaken according to PRISMA guidelines. A search strategy was performed using PubMed, Google Scholar, and Scopus for the period 2013–2023. All subjects were >18 years old, diagnosed with breast cancer, and received breast cancer therapies. Results: The most promising biomarkers that can be used for the development of an alternative risk cardiac stratification plan for the prediction and/or early detection of CTRCD in patients with breast cancer were identified. Conclusions: We highlighted the new insights associated with the use of currently available biomarkers as a standard of care for the management of CTRCD and identified potentially novel clinical biomarkers that could be further investigated as promising predictors of CTRCD.

## 1. Introduction

Despite therapeutic advancements in breast cancer, the use of certain types of breast cancer therapies can be associated with cardiac toxicity, a serious medical concern in oncology. The definition of cardiotoxicity due to anti-cancer treatment was previously provided in the 2016 European Society of Cardiology (ESC) Position publication and referred to any cardiovascular (CV) complication, which may include myocardial dysfunction and congestive heart failure (CHF), pericardial, valvular, or coronary artery diseases [1]. This definition was further refined in the most recent ESC guidelines in cardio-oncology by Lyon et al., 2022 [2]. Myocardial damage and HF due to chemotherapy have been associated with high rates of morbidity and mortality [3,4,5] and have attracted attention as a chemotherapy-associated CV complication. Anthracycline-based therapies and targeted therapies have been the most well-documented therapeutic compounds to be associated with CV toxicity [6,7,8,9,10,11,12].

Two categories of cancer therapy-related cardiac dysfunction (CTRCD) have been previously proposed depending on the effects of the chemotherapeutic agents on the pathophysiological and structural constant of the myocardium [13]. These include type I CTRCD, defined as permanent cardiotoxicity, typically induced by anthracyclines and distinctly characterized by cardiomyocyte injury, and type II CTRCD, which is considered reversible and mostly associated with the use of targeted therapy including the recombinant humanized monoclonal anti-HER2 antibody named trastuzumab [14]. However, this classification is currently debatable as a substantial recovery of cardiac function following anthracycline-induced cardiotoxicity can be achieved upon early diagnosis and prompt treatment [7,15]. In parallel, evidence suggests that type II CTRCD, which was proposed to be reversible, can persist for many years and may lead to irreversible cardiomyocyte apoptosis [16,17].

To improve the management of CTRCD, the ESC has published Clinical Practice Guidelines, which involve assessment of the left ventricular ejection fraction (LVEF), natriuretic peptides [e.g., brain natriuretic peptide (BNP)], and troponin-I determination [1,18]. Baseline risk stratification proformas have been recently proposed by the ESC in collaboration with the Cardio-Oncology Study Group, for patient stratification into severity groups based on the risk for CV complications prior to treatment [19]. Troponins, BNP and LVEF have been suggested as risk factors to be collected as part of the baseline proformas. The proposed risk stratification tool is expected to improve personalized approaches and mitigate the risk of cancer therapies-induced CV toxicity [19]. However, the management of CTRCD lacks clinical evidence, and therefore clinical practice is based on limited clinical studies. Prediction and better management of cardiotoxicity in patients receiving cytotoxic therapies are of critical importance. Reliable biomarkers that could predict cardiotoxicity and/or early onset of CTRCD are not currently available in the clinical setting.

It is clear that an ideal single biomarker for the prediction and/or detection of CTRCD cannot effectively assess and/or predict CTRCD [20]. While cardiac troponins and/or natriuretic peptides may be useful indicators of cardiomyocyte injury, their predictive capacity for the onset of cardiotoxicity still lacks reliability [18]. In addition, despite the fact that LVEF can be used as a strong indicator of cardiac dysfunction, it lacks sensitivity for early detection of subclinical cardiac impairment, which can otherwise be reversed upon prompt treatment [9,21,22]. Importantly, studies have shown that CHF, particularly in elderly individuals, can be associated with a normal LVEF [23]. Predictive biomarkers for the early and late onset of CTRCD are urgently needed to mitigate the risks associated with cardiac complications. Several clinical trials are currently ongoing, aiming to advance the diagnostic, monitoring, and predictive strategies of CTRCD in patients with breast cancer. Promising novel biomarkers related to cardiac function, inflammation, endothelial dysfunction, myocardial ischemia, and oxidative stress are currently under investigation [17,24,25,26,27]. This review aims to highlight the new insights into the use of existing and/or potentially promising novel serum and imaging biomarkers to be used as predictors and/or indicators of early CTRCD in patients with breast cancer. The time course of the assessment of such biomarkers will be also investigated.

## 2. Materials and Methods

### 2.1. Search Strategies

The systematic review was conducted based on the PRISMA 2020 Checklist [28]. The literature search was performed using the following electronic databases: Pubmed, Google Scholar, and Scopus with the aim to identify new insights on the potential of using existing clinical and/or novel biomarkers as predictive and/or diagnostic indicators of CTRCD in patients with breast cancer. In addition, the reference lists of relevant articles were searched. Studies published between 2013–2023 were included in the review. Restrictions on the search included studies written in English language, gender, and human studies. There were no restrictions on the geographics. The search strategy was conducted using the following keywords: (cardiotoxicity) OR (induced cardiotoxicity) OR (chemotherapy-induced cardiotoxicity) OR (therapy-induced cardiotoxicity) OR (breast cancer therapy-induced cardiotoxicity) AND (breast cancer) OR (breast carcinomas) AND (biomarkers) OR (clinical biomarkers) OR (circulating) OR (predict*). This search resulted in a total of 2229 results for the period of interest (2013–2023) from the three databases searched. The search strategy developed was peer-reviewed by an experienced information specialist using the Peer Review of Electronic Search Strategies (PRESS) checklist [29].

**Figure 1:**
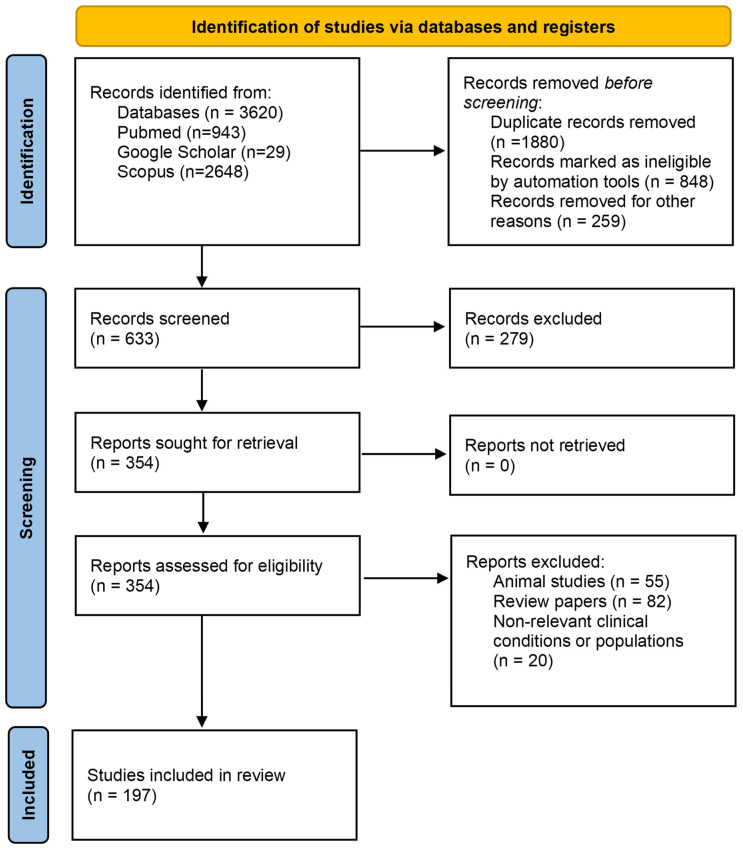
PRISMA 2020 flow diagram for the systematic review [28].

A second search was also conducted using an extensive list of keywords specific to emerging cardiac biomarkers for the detection of CTRCD. The keywords are the following: (novel biomarkers) OR (inflammatory) OR (endothelial) OR (oxidative stress) OR (fibrosis) OR (angiogenesis) OR (interleukin 16) OR (interleukin 1) OR (galectin-3) OR (N-terminal pro-B-type natriuretic peptide) OR (B-type natriuretic peptide) OR (C-reactive protein) OR (troponin) OR (high-sensitivity cardiac troponin) OR (hs-cTn) OR (hs-cTnI) OR (microRNA) OR (miRNAs) OR (PIGF) OR (placenta growth factor) OR (ST2) OR (growth differentiation factor 15) OR (GDF-15) OR (myeloperoxidase) OR (MPO) OR (contractility) OR (cardiac injury biomarkers) OR (biomarkers) OR (clinical biomarkers) OR (glycogen phosphorylase BB) OR (GPBB) OR (left ventricular diastolic dysfunction) OR (left ventricular ejection fraction) OR (LVEF) OR (left ventricular global longitudinal strain) OR (GLS) OR (myoglobin) OR (heart-type fatty acid-binding protein) OR (lipopolysaccharide-binding protein) OR (myocardial cell apoptosis) OR (arginine) OR (oxide metabolite) OR (fms-like tyrosine kinase receptor) OR (sFlt-1) OR (thrombin–antithrombin complex) AND (cardiotoxicity) OR (chemotherapy-induced cardiotoxicity) OR (induced cardiotoxicity) OR (therapy-induced cardiotoxicity) OR (cardiomyopathy) OR (heart failure) OR (cardiac dysfunction) AND (breast cancer) OR (breast carcinomas) AND (biomarkers) OR (clinical biomarkers) OR (circulating) OR (predict*). This search resulted in a total of 1391 papers. Combining the search results using the two keyword lists, a total of 3620 papers were obtained, of which 1880 papers were duplicates. The duplicates were removed using the Endnote reference management software. A total of 259 were excluded during the prescreening stage based on the relevance of the title. A total of 633 papers were selected for screening, of which 279 papers were excluded based on the relevance of the abstract. The remaining 354 papers were assessed based on the eligibility criteria and 157 papers were excluded for the reasons mentioned in Figure 1. A total of 197 papers were included in this review (Figure 1). Two reviewers (A.A. and A.C.) conducted the screening process independently and identified the relevant studies that meet the eligibility criteria. Any discrepancies were resolved by discussion.

### 2.2. Study Population

The study eligibility criteria were set up based on the participants, intervention, comparator, and outcomes (PICO) elements of the review question. The eligible studies meet the following inclusion criteria: (1) female patients aged >18 years old that have been diagnosed with breast cancer at any disease stage, (2) received breast cancer therapies, (3) performed cardiac examination at the baseline. Exclusion criteria included: patients that received cardiotoxic therapy for the treatment of secondary malignant neoplasm. Outcome measures: cardiac examination performed to determine changes in cardiac function at the baseline and at follow-ups. The association of existing and/or novel biomarkers in the prediction and/or detection of CTRCD was evaluated.

### 2.3. Selection Criteria

Randomized controlled trials (RCTs), other clinical trials, cohort studies, and post hoc analyses were included in the review. Literature reviews, conference abstracts, and posters/abstracts were excluded. Preclinical reports/animal studies were not included as they were beyond the scope of this review. Quality assessment of the clinical trials was conducted by two reviewers independently according to the CASP randomized controlled trial standard checklist. Risk-of-bias assessment was performed using the Robvis tool [30]. Five domains were assessed including: bias arising from the randomization process; bias due to deviations from intended intervention; bias due to missing outcome data; bias in measurement of the outcome; bias in selection of the reported result. Studies were excluded when the outcomes of interest were not measured, or a particular outcome was explicitly not included in the measurement. Studies with no definition of cardiotoxicity and no treatment with breast cancer therapies were excluded from the review.

### 2.4. Data Extraction

Data extraction was performed by two reviewers (A.A. and A.C.) independently using an electronic custom-built structural data collection form. Details on the methodology, study design (e.g., multicenter study) characteristics of the control and intervention groups, outcome measures (e.g., LVEF measurements, circulating biomarkers, time points assessed), and follow-up duration were extracted.

### 2.5. Data Synthesis

The data synthesis approach was decided based on the selected clinical studies. The outcome measures, similarities (if any) in the study design, and the data available in each study were considered for the synthesis method. Patients with CV diseases and/or elevated cardiac biomarkers (if measured) at the baseline as well as patients with metastatic breast cancer at the baseline were treated as subgroups if an adequate number of trials were available. According to the clinical studies selected, the following data synthesis was applied: summarizing effect estimates, providing statistical outcomes (e.g., *p* values), and confidence intervals when available. In the case where synthesis was considered to be inappropriate, a structured reporting of effects was applied, and the most relevant and trustworthy studies were prioritized. A graphical abstract was also created (BioRender).

## 3. Results

Anthracyclines (e.g., doxorubicin, epirubicin) and targeted therapies such as trastuzumab have been the most documented therapeutic compounds to be associated with CV toxicity [6,7,8,9,10,11,12]. However, other breast cancer therapies that can affect the CV system include alkylating agents (e.g., cyclophosphamide), VEGF inhibitors (e.g., bevacizumab), tyrosine kinase inhibitors (e.g., lapatinib, neratinib), antimicrotubular agents (e.g., paclitaxel, docetaxel), antimetabolites (e.g., fluorouracil, capecitabine) and hormone therapies including cyclin-dependent kinases (CDK) 4/6 inhibitors (e.g., palbociclib, ribociclib, abemaciclib) [16]. Aromatase inhibitors (e.g., anastrozole, letrozole) have not been associated with induced CV death, but an association with angina and hypertension has been identified. In addition, venous thromboembolism has been linked with the use of tamoxifen, a blocker of estrogen receptor [31,32]. Radiation therapy can increase the risk of cardiac dysfunction in patients with breast cancer [33]; however, radiation-induced cardiotoxicity is beyond the scope of this review. This review aims to provide new insights into the use of existing and/or potentially novel blood and imaging biomarkers to predict the incidence of cardiotoxicity in patients with breast cancer during cardiotoxic therapies. The time course of the assessment of the biomarkers during treatment was also reported.

### 3.1. Cancer Therapy and Cardiotoxicity

#### 3.1.1. Chemotherapy

Anthracycline-induced cardiotoxicity (AIC) was shown to be cumulative dose-related and is categorized into three distinct groups according to the most recent classification: the acute onset, which occurs following a single dose/course and symptoms appear 14 days after treatment completion, which is usually reversible; the early onset of chronic cardiotoxicity, which occurs within one year and can progressively lead to CHF; and the late-onset of chronic cardiotoxicity, which progresses for years after completion of treatment (median of 7 years post-treatment) and resembles chronic cardiac failure [1,34]. Acute onset of AIC is a rare event and is observed in approximately 5% of patients. It manifests with electrocardiographic (ECG) changes in 20–30% of the patients, supraventricular arrhythmias, acute myocarditis with cardiomyocyte injury, acute CHF, and/or pericarditis. Despite the extensive research on the mechanism through which anthracyclines lead to cardiotoxicity, the exact molecular pathogenesis of AIC remains elusive. The acute onset of cardiotoxicity can be characterized by reduced cardiac mass via p53-dependent inhibition of the mammalian target of rapamycin (mTOR) signaling [35], induction of oxidative stress [36], and topoisomerase inhibition resulting in double-strand breaks [37]. However, novel AIC pathways are continuously emerging [37,38].

For example, in the case of doxorubicin, studies demonstrated doxorubicin-induced ferroptosis, which involves the iron-dependent formation of lipid peroxides [39], induction of cell death via necroptosis [40] and/or induction of pyroptosis through upregulation of the terminal differentiation-induced non-coding RNA (TINCR) followed by activation of the NLR family pyrin domain containing 3 (NLRP3)-caspase 1 pathway [38,41,42]. In addition, preclinical evidence suggests that the induction of death receptors associated apoptosis by anthracycline agents (daunorubicin, idarubicin, and epirubicin) as demonstrated in human induced pluripotent stem cells-derived cardiomyocytes (iPS-CMs) is a potentially critical mechanism that may underly the cardiotoxic potential of these agents [42].

Other cardiotoxic breast cancer therapy includes DNA alkylating agents (e.g., cyclophosphamide). Specifically, cyclophosphamide can induce hemorrhagic myocarditis, particularly at high doses, even though lower doses of cyclophosphamide have been associated with CHF and/or pericardial effusion. The cardiotoxic incidences of cyclophosphamide have been attributed to its metabolites, which have been suggested to promote oxidative stress resulting in endothelial capillary damage, hemorrhage, edema, and thrombosis [43,44]. Combination regimen schemes composing taxanes (e.g., docetaxel, paclitaxel), anthracycline, and cyclophosphamide were associated with a higher risk of cardiotoxicity [45,46]. However, large-scale high-quality clinical studies are needed to further assess the cardiotoxic outcomes of breast cancer combination treatment regimens [45].

#### 3.1.2. Targeted Therapy

The human epidermal growth factor receptor 2 (HER2) is a transmembrane glycoprotein [47,48], shown to be overexpressed in 15–20% of breast cancers and conferring worse prognosis [49,50,51,52,53,54,55,56]. Trastuzumab, a humanized monoclonal antibody, is the most frequently used HER-2 targeted therapy for the treatment of HER2+ breast cancer contributing to markedly improved survival rates [57,58,59]. Other HER2-targeted drugs used for breast cancer include pertuzumab and the most recent agents, tucatinib and trastuzumab deruxtecan [57,58,59].

Despite the therapeutic impact of HER2-targeted therapy [60,61,62], unexpected cardiotoxicity can interfere with their efficacy [63]. Zhang et al., 2022 [64], have demonstrated that trastuzumab or trastuzumab in combination with pertuzumab, resulted in decreased LVEF by at least 10% in 15.9% of the patients (67 out of 420) with the incidence of 14.3% and 17.9%, respectively. Contrary to AIC, trastuzumab-induced cardiotoxicity manifests as an asymptomatic drop in the LVEF followed by infrequent CHF and in rare cases cardiac death [65,66,67]. The risk of cardiac dysfunction increases in patients that receive anthracyclines plus cyclophosphamide followed by trastuzumab (27%) [67].

Trastuzumab-related cardiotoxicity can be reversible upon discontinuation of treatment [5], which may, however, be associated with tumor recurrence and worse overall survival [68,69]. Evidence also supports that the recovered LVEF measurements in patients treated with trastuzumab for 12 months (48.53%), never reached the baseline LVEF levels at 30 months after treatment completion [70]. Calvillo-Argüelles et al., 2020 [5], revealed that 43% of patients (10 out of 23) of HER2+ metastatic breast cancer patients, had their treatment with trastuzumab interrupted. However, only 30% of patients had a cardiological examination while 17% received cardioprotective therapy suggesting a potential gap in cardiac care. Even though the mechanism by which trastuzumab exhibits its cardiotoxic effect is unclear, it is thought to be associated with the direct target of the HER2/neu, which is also expressed on cardiomyocytes and was shown to possess a cardioprotective role. Preclinical studies demonstrated that ErbB2 knockout mice (ErbB2-CKO) showed poor survival and dilated cardiomyopathy whilst cardiomyocytes derived from ErbB2-CKO mice had increased susceptibility to the fatal cell damage induced by anthracyclines [71]. It is worth mentioning that not all anti-HER2 therapies exhibit similar cardiotoxic potential. For example, pertuzumab and lapatinib have much less severe cardiotoxicity profiles compared to trastuzumab [72,73].

### 3.2. Traditional Biomarkers

#### 3.2.1. Troponins

Cardiac troponins are regulatory protein complexes in the skeletal and cardiac muscle, responsible for cardiac muscle contractions. Cardiac troponin T (cTnT), cardiac troponin I (cTnI), and troponin C are the three subunits of troponins, exclusively found in the myocardial tissue. Systemic troponin release upon cardiomyocyte necrosis is indicative of myocardial infarctions [74,75]. Consequently, troponins are established diagnostic biomarkers for the detection of acute and chronic myocardial damage [75,76]. Cardiac troponins are considered to be amongst the cornerstones of cardiotoxicity monitoring in patients treated with cardiotoxic therapies [77,78,79,80]. Previous studies revealed troponins as predictive biomarkers of trastuzumab-induced cardiotoxicity in breast cancer patients [80]. However, analytical sensitivity can differ between cTnI and cTnT by 10-fold, and cTn values may vary between different assay generations and instruments [81,82]. In addition, the time course for the detection of induced troponin elevation in response to cardiotoxic anti-cancer treatment is variable and not clearly understood compared to the changes mediated by conventional CV events [83]. Clinical studies have investigated the utility of troponins in predicting and/or detecting CTRCD in breast cancer patients.

Shafi et al., 2017 [84], showed that the elevation of cTnI (*p* < 0.001) detected after one cycle of anthracycline-based chemotherapy was a frequent event in patients experiencing cardiotoxicity (6 out of 82 patients, 7%). cTnI proved a strong independent predictor of the incidence of cardiotoxicity (95% CI (0.003546–0.2535), *p* < 0.001]) and the failure of LVEF recovery (95% CI (0.002484 to 1.680)). Cardiotoxicity was defined as a drop in LVEF of ≤10% from baseline or a decline in LVEF < 50% and measured before treatment and every 3 months during the first year of treatment with anthracyclines. A total of 10 cardiac events (LVEF reduction, CHF, acute coronary syndrome, arrhythmias) occurred during the study, 9 of which were associated with high levels of cTnI 9 (*p* < 0.001). Limitations of this study include the small sample size, the inability to assess delayed cardiotoxicity due to the short follow-up, and the recruitment of patients from a single cancer center.

Another study [10], revealed similar results showing that elevated baseline levels of cTnI (>40 ng/L) in 13.6% (56 of 412) and cTnT (>14 ng/L) in 24.8% of (101 of 407) HER2+ breast cancer patients were associated with a significantly higher risk of LVEF decline (hazard ratio (HR) of 4.52, 95% CI (2.45–8.35), *p* < 0.001 and 3.57; 95% CI (1.95–6.55), *p* < 0.001 in the univariate model, respectively) in response to trastuzumab treatment. For 31 patients, an increase in cTnI (n = 6) and cTnT (n = 25) occurred during treatment. Primary cardiotoxicity was defined as the clinical manifestation of CHF New York Heart Association (NYHA) class III or IV and LVEF drop by at least 10% from baseline or decline of LVEF < 50%. The secondary cardiac endpoint was defined as an asymptomatic or mildly symptomatic decline in LVEF.

The predictive value of high sensitivity TnT (hscTnT) was highlighted by Blaes et al., 2015 [85], showing that patients with elevated levels of hscTnT (2.7 pg/mL, *p* = 0.07) at baseline were at higher risk of LVEF decline (n = 12, median LVEF = 54%) compared to the patients with no changes in LVEF (n = 6, median LVEF = 64%, 0.1 pg/mL), suggesting its utility as a predictive biomarker of anthracycline-induced cardiotoxicity. This is in contrast to the cTnI and cTnT, which were undetectable at baseline. A Spearman correlation revealed a trend towards greater reduction in LVEF in patients with increased levels of hscTnT levels at baseline (−0.54, 95% CI (−0.80 to −0.08), *p* = 0.02) and creatine kinase-MB (CK-MB) (−0.49, 95% CI (−0.77 to −0.01) *p* = 0.04). However, the study included a small sample size, which was not sufficient to allow for the detection of potentially asymptomatic drops of LVEF.

A progressive increase in hscTnT was noted in a prospective study of 72 breast cancer patients in response to anthracycline chemotherapy reaching maximum levels (from baseline 4.6 ± 2.1 ng/L to 15.7 ± 7.4 ng/L) at 96 ± 13 after treatment initiation [86]. Interestingly, levels of hscTnT were slightly increased in patients that did not experience cardiotoxicity (4.8 ± 2.1 vs. 3.1 ± 0.2; *p* = 0.006) and therefore no statistically significant differences between patients with cardiotoxicity (n = 7) versus patient without (n = 65) were observed either at baseline or during treatment. The authors also showed that hscTnT (odds ratio (OR) = 0.923, 95% CI (0.780–1.042), *p* = 0.27) could not predict CTRCD in these patients. In particular, higher levels of hscTnT were noted in 62.5% of the patients even though cardiotoxicity was noted only in 9.7% of the patients. It was also shown that troponin levels were linearly correlated with age (*p* < 0.001, R^2^ = 0.16) suggesting that age should be also considered when assessing hscTnT levels. Limitations of this study include the fact that the patients were relatively young, had a mean age of 52.0 ± 9.8 years old, and hence data cannot be extrapolated to older patients. In addition, a limited number of patients experienced cardiotoxicity and patients were recruited from a single center suggesting the need for additional larger cohort studies.

In another study of 134 female breast cancer patients, treated with doxorubicin (DOX) and epirubicin, a six-fold increase in hscTnI was noted in breast cancer patients (61%) after treatment (38.8 ± 26.7 vs. 7.0 ± 4.1 ng/mL, *p* < 0.0001) compared to baseline. However, there was a poor agreement between changes in troponin and cardiotoxicity (Kappa −0.017, *p* = 0.67) and no association with changes in LVEF or GLS (R^2^ = 0.03 and 0.04, respectively) [87]. Cardiotoxicity was defined as previously described by the European Society of Medical Oncology (ESMO) [88]. Limitations of the study included the small sample size and single-center patient recruitment, which limit the extrapolation of the findings.

In contrast, Pillai et al., 2022 [89], showed significantly increased levels of cTnI at 6 months and cTnT at 3 and 6 months of trastuzumab treatment (with or without pertuzumab) in combination with paclitaxel or docetaxel (with or without carboplatin) in HER2-positive breast cancer patients (n = 17, *p* < 0.05) compared to the healthy controls (n = 17). However, the findings need to be validated by larger cohort studies and for a longer follow-up period. It is worth noting that a combination with taxanes, which may also contribute to cardiotoxicity, may interfere with trastuzumab-induced cardiotoxicity.

All studies retrieved through this review in relation to the role of troponins in predicting and/or detecting CTRCD in breast cancer patients [10,83,84,85,86,87,89,90,91,92,93,94,95,96,97,98,99,100,101,102,103,104,105,106,107,108,109,110,111,112,113,114,115,116,117,118,119] are shown in Table S1.

#### 3.2.2. Natriuretic Peptides (NPs)

Natriuretic peptides (brain natriuretic peptide (BNP) and N-terminal part of the pro-peptide of BNP (NT-proBNP)) are cardiac hormonal excretions from ventricular cardiomyocytes [120,121]. Several studies reported that increased levels of BNP or NT-proBNP in the serum are an indicator of CHF [120,121]. Clinical studies have investigated the correlation of BNP and NT-proBNP with breast cancer therapy-induced CV events resulting in inconsistent results.

A prospective study of 136 HER2+ breast cancer patients showed patients experiencing trastuzumab-induced cardiotoxicity had lower baseline LVEF (n = 6, LVEF 57.08 ± 1.36%) compared to the control (n = 125, LVEF 61.42 ± 0.26%) in response to trastuzumab treatment [122]. In addition to reduced baseline LVEF, patients who experienced CTRCD had a three-fold increase in NT-proBNP (from 198.8 ± 64.0 pg/mL to 678.7 ± 132.4 pg/mL; *p* < 0.05) compared to the control showed a reduction at month 6 (from 131.2 ± 20.9 pg/mL to 86.7 ± 8.8 pg/mL; *p* < 0.05). Six out of a total of one-hundred and thirty-six patients (4.4%) experienced CTRCD at 6 or 12 months of trastuzumab treatment. The authors proposed that assessing changes in NT-proBNP could potentially replace echocardiographic examination during the one year of trastuzumab therapy. The authors calculated the δNT-proBNP which is defined as the average difference between the baseline NT-proBNP to 6 months and from baseline to 12 months [122]. They suggested 75.8 pg/mL as the cut-off value for δNT-proBNP to identify patients for echocardiographic assessment. In addition, the authors highlighted that δNT-proBNP is more suitable since absolute NT-proBNP values at baseline differ between healthy individuals.

In line with a previous prospective study are the results of a retrospective observational study of a total of 66 HER2+ breast cancer patients showing high levels of NT-proBNP (OR = 22.0, 95% CI (5.7–85.4); *p* < 0.0001) in patients who experienced cardiotoxicity during trastuzumab therapy (18 out of 66 patients, 27.3%) with a strong association with diabetes mellitus (OR = 5.9, 95% CI [1.2–28.5]; *p* = 0.028) as revealed by a binary logistic regression analysis [123]. A significant elevation of NT-proBNP was noted at 3, 6, and 12 months of trastuzumab treatment compared to baseline. In addition, a significant association between LVEF and NT-proBNP was noted in women ≥ 50 years old compared to women < 50 years old. Cardiotoxicity was defined as previously described in the herceptin adjuvant (HERA) trial [4] and involved LVEF decrease with the development of CHF or LVEF decline ≥ 10% resulting in an asymptomatic drop in LVEF < 50%. It must be highlighted that the baseline levels of NT-proBNP were not available in this study, which did not allow for assessing the predictive role of baseline NT-proBNP in patients who developed CTRCD during trastuzumab treatment. A small number of participants were included in this study, resulting in a small size of subgroups of 18 patients treated with anthracyclines and 3 patients treated with taxanes (docetaxel or paclitaxel) whilst 8 patients were affected by diabetes, which, however, showed significant association with CTRCD.

Bouwer et al., 2019 [124], detected higher levels of NT-proBNP in patients who experienced CTRCD (16.8 pmol/L; *p* = 0.031) during trastuzumab treatment compared to patients with no cardiotoxicity (10.1 pmol/L). An increase in NT-proBNP at any time point during follow-up was associated with a decline in LVEF (95% CI (−2.2%, –6.7%); *p* < 0.001). In addition, patients who developed CTRCD had statistically higher baseline NT-proBNP levels (+10.2 pmol/L) compared to those without (+ 2.5 pmol/L) and it was related to the occurrence of CTRCD during follow-up (HR = 1.04, 95% CI (1.02–1.07; *p* = 0.003). Despite the significant results showing the potential of using NT-proBNP to identify patients with a higher risk of CTRCD, the authors concluded that the NT-proBNP is not a surrogate diagnostic tool for the detection of the early onset of CTRCD due to the fact that there was no gradual and/or sudden increase in the levels of NT-proBNP prior to the development of CTRCD. In the study by Alves et al., 2021 [125], elevated levels of NT-proBNP by 2.1-fold were detected in patients with cardiotoxicity (116.55 ± 107.66 pg/mL) at 7 days after the last infusion with doxorubicin compared to the baseline (54.51 ± 28.58 pg/mL; *p* < 0.05). The variability of results observed in the studies might be attributed to the different therapeutic regimens, the sample size, the time points assessed, the definition of cardiotoxicity used in each study, and/or the different assays used to detect NT-proBNP as supported by Bouwer et al., 2019 [124].

Matos et al., 2016 [126], included a total of 92 breast cancer patients of which 20.6% had a drop in LVEF ≥ 10% and paradoxically had significantly higher LVEF (70.7 ± 4.4%, *p* = 0.0002) at baseline compared to the patients with no LVEF decline (64.8 ± 5.5%). All patients were pre-treated with anthracyclines prior to trastuzumab treatment, and 82 patients (89.1%) received taxanes. In this study, baseline NT-proBNP was not significantly associated with trastuzumab-related LVEF reduction and remained within the normal range (<300 pg/mL) during the follow-up time points (4, 8, and 12 months during trastuzumab).

In contrast, Lu et al., 2019 [127], in 149 breast cancer patients, showed significantly increased levels of BNP in patients with cardiotoxicity during anthracycline treatment compared to the non-cardiotoxicity group and it was an independent predictor of anthracycline-related cardiac dysfunction (*p* = 0.047). An increase in BNP was noted after each treatment course. The baseline serum BNP levels did not predict the onset of cardiotoxicity in patients. Cardiotoxicity was defined as a drop in LVEF < 55, a reduction in LVEF ≥ 10%, and an increase in LVDs ≥ 7 mm from baseline. Despite the significant results, the study included a small sample size, which may have resulted in a limited number of patients with cardiotoxicity. It is worth noting that other risk factors including diabetes were not considered.

Kouloubinis et al., 2015 [128], demonstrated a significant drop in LVEF (*p* < 0.001) in metastatic breast cancer patients (n = 26) after treatment with epirubicin and paclitaxel associated with elevated levels of NT-proBNP (158.0 ± 8.4 vs. 283.3 ± 27.2 pre- and post-treatment, respectively, *p* < 0.001) and sFas (308.8 ± 65.1 vs. 517.8 ± 91.0 pre- and post-treatment, respectively, *p* < 0.001) as well as with a decrease in sFasL (86.6 ± 12.6 vs. 47.9 ± 8.4 pre- and post-treatment, respectively, *p* = 0.010) post-treatment. Congestive HF was noted in two of the metastatic patients (7.6%) at 12 and 14 months post-treatment. The authors have introduced the apoptotic markers sFas, sFasL, and the cardiac-specific marker NT-proBNP as promising markers for the early detection of CTRCD in breast cancer patients. However, larger prospective studies are needed to further validate their clinical utility.

The studies focusing on the role of natriuretic peptides (NPs) in predicting and/or detecting CTRCD in breast cancer patients [86,87,90,91,92,96,97,98,99,100,101,103,105,106,109,113,114,117,118,119,122,123,124,125,126,127,128,129,130,131,132,133,134,135,136,137,138,139,140,141,142,143,144,145], as identified in the review, are shown in Table S1.

#### 3.2.3. Left Ventricular Ejection Fraction (LVEF) and Strain Changes

The role of left ventricular ejection fraction (LVEF) as a biomarker of diagnosis and prediction of CTRCD has been presented in a number of studies (Table S1). Further to studies demonstrating the role of LVEF in assessing and/or predicting CTRCD [118,146], Stoodley et al., 2013 [147], revealed a significant reduction in global systolic strain (GSS) (myocardial imaging) in HER2-negative breast cancer patients promptly after (−19.0 ± 2.3% to −17.5 ± 2.3%; *p* < 0.001) and at 6 months of anthracycline therapy −8.2 ± 2.2%; *p* = 0.01). On the contrary, no changes were noted in the LVEF at either time point. Even though global systolic strain stabilized by month 12 in most patients, 16% of the patients (n = 8) who received higher doses of anthracyclines and had greater myocardial systolic dysfunction at month 6 (≤−17.2%) continued to have reduced global systolic strain.

A recent publication in the *Journal of the American Heart Association* [148], measured changes in LV end-diastolic volume (LVEDV), LV end-systolic volume (LVESV), global circumferential strain (GCS) and LVEF at baseline and at 3 and 24 months after treatment initiation in a prospective cohort study of 95 patients diagnosed with breast cancer (n = 29), soft tissue sarcoma (n = 5) or lymphoma (n = 37). Patients received anthracyclines (n = 48), trastuzumab (n = 2), taxanes (n = 28), cyclophosphamide (n = 49), other chemotherapy (n = 45), and immunotherapy (n = 23). Cardiac toxicity was defined as a drop in LVEF by >5% or LVEF by < 50% from baseline to 24 months. A decline in LVEF was noted during the 24 months (from 62 ± 7% to 58 ± 9%; *p* < 0.0001), with 42% of patients showing an LVEF drop by >5% at 24 months. The authors identified predictive factors at baseline or at 3 months of treatment for a persistent 2-year decline of LVEF > 5%. These included an increase in LVESV (>3 mL; *p* = 0.033) or a drop in LVEDV by >10% with a mild change in LVESV (*p* = 0.001), or a GCS increase by >10% with an increase in LVESV > 3 mL or increased GCS by >10% with a mild change in LVESV (<3 mL) with a drop in LVEDV by > 10 mL. It is worth mentioning that all logistic regression models remained significant after accounting for the CV risk factors, chemotherapy regimens received, demographics, sex, age, body mass index, administration of cardioactive substances, and cancer types (*p* = 0.001 to *p* = 0.037). A reduction in LVEF of >5% was characterized by the authors as a subclinical decline in LVEF and further studies are required to address the long-term effects in patients that receive cardiotoxic therapies. It is important to note that one of the limitations of the study is that a total of 24 patients did not manage to complete the 2-year follow-up monitoring, which may have introduced bias in the analysis of the results. It was noted that the cancer type was a contributing factor to the discontinuation of the patient participation (*p* = 0.0004); however, breast cancer patients (88%) had a higher likelihood to complete the study compared to the patients with sarcoma or lymphoma. In addition, the small sample size did not allow for the detection of a larger decline in LVEF that would lead to CHF.

In another study by Bulten et al., 2015 [149], evaluating ^123^I-metαiodobenzylguanidine (^123^I-mIBG) scintigraphy and potential association with conventional ECHO parameters, showed that global radial strain (GRS) demonstrated the strongest association with delayed heart/mediastinum (H/M) ratio (Pearson’s r 0.36; *p* = 0.01), in patients after 1 year of treatment with anthracycline (docetaxel, doxorubicin, and cyclophosphamide).

A prospective study by Tahir et al., 2021 [150], with a total of 66 breast cancer patients (53 ± 13 years), showed that amongst the total of 39 patients, who received epirubicin-based chemotherapy, 20% (n = 8), developed therapy-related cardiac dysfunction. Increased myocardial T1 relaxation time predicted the onset of cardiac dysfunction after therapy completion (follow-up 1) with an area under the curve (AUC) of 0.712 (95% CI (0.587–08.16); *p* = 0.005), 100% sensitivity but lower specificity of 44% (31–58%). A combination of elevated T1 and reduced LVEF ≤ 60% after treatment completion resulted in high sensitivity of 78% (44–95%), improved specificity of 84% (72–92%), and AUC of 0.810 (0.695–0.896). Cancer therapy-related cardiac dysfunction was defined as a decline in LVEF ≥ 10% resulting in LVEF < 55% or GLS change by >15% at the second follow-up (13 ± 2 months after treatment). Similarly to the other studies, a small sample size is a limitation and so the largest cohort studies are needed to better define the role of the cardiac magnetic resonance imaging (CMR) parameters in the prediction of therapy-induced cardiac dysfunction. Wang et al., 2020 [151], conducted a prospective study to assess the utility of a relatively new technology in monitoring cardiac function, called three-dimensional speckle tracking imaging (3D-STI), which is a combination of two-dimensional speckle tracking imaging (2D-STI) and real-time three-dimensional echocardiogram (RT-3DE). The study included a total of 64 breast cancer patients and showed that right ventricular global longitudinal strain (RVGLS) and right ventricular global area strain (RVGAS) were significantly reduced after chemotherapy (−26.10% ± 2.33% and −35.78% ± 5.60%) compared to the baseline (*p* < 0.05) in the patients that received pirarubicin chemotherapy with dexrazoxane (−28.60% ± 2.57% and −38.66% ± 5.73%). Dexrazoxane is an anti-neoplastic agent that can be used to reduce the risk of anthracycline-related CHF and LVEF impairment via inhibiting topoisomerase 2β [152]. The 3D-STI examination showed that RVGLS and RVGAS were significantly (*p* < 0.05) higher in the patients treated with chemotherapy plus dexrazoxane (−26.10% ± 2.33% and −35.78% ± 5.60%, respectively), versus the control group, that did not receive dexrazoxane (−24.59% ± 2.36% and −32.77% ± 6.23%, respectively). Overall, this study highlights that monitoring the functional changes in the right ventricular myocardium during chemotherapy in post-operative breast cancer patients using 3D-STI can provide an early and accurate indication of potential chemotherapy-induced cardiac dysfunction.

A study assessing an extended panel of pro-inflammatory biomarkers in breast cancer survivors revealed that LVEF was lower in patients that received chemotherapy at least 5 years ago (57.5% (55.0–60.0%); *p* < 0.001), compared to the healthy individuals that have not been diagnosed with breast cancer (59.0% (57.0–62.0%) [153]. Lower LVEF was significantly associated with higher levels of pro-inflammatory genes. This may suggest that breast cancer survivors treated with chemotherapy may experience a potentially prolonged and persistent pro-inflammatory state [153].

According to the 2022 ESC Guidelines [2], in addition to the measurement of cardiac serum biomarkers and echocardiography, cardiac imaging techniques such as cardiac magnetic resonance (CMR) allow for the early detection of CTRCD [154]. Cardiac imaging should be performed for any patient with symptoms of cardiac dysfunction following cardiotoxic cancer therapy. Myocardial injury as indicated by cardiac magnetic resonance imaging (cMRI) in patients was found to be correlated with elevated concentrations in cTnI, however, small myocardial infarcts (<1 g) are undetectable [74]. Multimodal imaging using stress echocardiography, chest computed tomography (CT), positron emission tomography (PET) imaging, and stress CMR are suggested as excellent imaging tools for the diagnosis of ischemic incidences in patients treated with cardiotoxic cancer therapies [155]. GLS and three-dimensional (3D)-LVEF (transthoracic echocardiography (TTE)) are recommended for the diagnosis of asymptomatic CTRCD [155,156]. In particular, GLS assessment can indicate the presence or absence of asymptomatic myocardial injury in patients with physiologically low levels of LVEF [157]. In addition, the predictive capacity of tissue velocity imaging (TVI) and STI in LV dysfunction was demonstrated in breast cancer patients treated with epirubicin [158]. Overall, the contribution of troponins, NT-proBNP or BNP, and LVEF and associated parameters in detecting and monitoring CTRCD is undoubtable, but additional studies are required in order to define their exact roles to predict CTRCD. The studies focusing on the role of LVEF, strain changes, and other imaging biomarkers in predicting and/or detecting CTRCD in breast cancer patients [84,91,94,95,99,108,109,113,118,119,130,131,132,133,137,139,141,142,146,147,148,149,150,151,153,158,159,160,161,162,163,164,165,166,167,168,169,170,171,172,173,174,175,176,177,178,179,180,181,182,183,184,185,186,187,188,189,190,191,192,193,194,195,196,197,198,199,200,201,202,203,204,205,206,207,208,209,210,211,212,213,214,215,216,217,218,219,220], as identified in the review, are shown in Table S1.

### 3.3. Emerging Biomarkers

#### 3.3.1. Genetic Susceptibility to CTRCD

##### SNPs in HER2/neu

Several studies have identified genetic variants as risk factors associated with higher incidences of cardiotoxicity induced by breast cancer therapeutic regimens involving trastuzumab or anthracyclines [221,222,223,224,225]. Such genetic variations possess a predictive value indicating the patients are susceptible to CTRCD. Single nucleotide polymorphisms (SNPs) are common genetic variations, that have the potential to affect response to treatment and influence therapy-induced cardiotoxicity. A recent search on the Exome Variant Server (https://evs.gs.washington.edu/EVS/ (accessed on 16 March 2023) reveals a total of 67 missense SNPs in the *ERBB2* gene; however, their role in the therapeutic efficacy and response in patients with HER2 breast cancer remains to be elucidated.

To date, the best-documented polymorphism is at the amino acid position 655 of the *HER2*/*neu* (*HER2* lle655Val), which results in the nucleobase change in adenine to guanine (*HER2* codon 655 A>G), which in turn changes the translation of the amino acid isoleucine to valine (Ile/Val). Five prospective studies [221,222,226,227,228] and one retrospective study [229] have investigated the association of *HER2* codon 655 AG polymorphism with trastuzumab-induced cardiotoxicity leading to contradictory results (Table S2).

Four out of the six studies highlight the significant correlation of the *HER2* codon 655 AG polymorphism with cardiotoxicity induced by trastuzumab-based therapies [221,227,228,229]. Tan et al., 2020 [221] showed that the incidence of cardiotoxicity in response to epirubicin/cyclophosphamide followed by docetaxel and trastuzumab was higher in patients that harbor the *HER2* codon 655 AG genotype (24 out of 91 patients, 26.4%) compared to the patients with *HER2* codon 655 AA genotype (64 out of 91 patients, 70.3%) (*p* = 0.017). Cardiotoxicity was defined as a reduction in LVEF by at least 10% from the baseline resulting in LVEF < 53 and/or manifestation of CHF, fatal arrhythmia, or acute coronary artery syndrome. The minority of the patients in this study (3 out of 91, 3.3%) had the *HER2* codon 655 GG genotype, which was associated with a higher incidence of cardiotoxicity compared to the *HER2* codon 655 AG genotype but without statistical significance (*p* = 0.496), probably due to the small sample size. Univariate logistic regression analysis showed that the *HER2* codon 655 AG (OR = 3.117, *p* = 0.008), baseline cTnI (OR = 1.030, *p* < 0.001), and baseline NT-proBNP levels (OR = 1.015, *p* = 0.010) were correlated with cardiotoxicity. However, no clear association is provided between each cardiotoxic parameter used to define cardiotoxicity. This is except, for LVEF, which was similar in patients with or without the *HER2* codon 655 AG polymorphism, suggesting no correlation.

On the contrary, Roca et al., 2013 [227], revealed that patients with *HER2* codon 655 AG experienced significantly increased CV complications; that is LVEF < 50%, compared to the patients with the homozygous allele (*HER2* codon 655 AA) (69% and 31%, respectively; 95% CI (1.11–13.8), *p* = 0.025) in response to trastuzumab therapeutic regimen.

The minority of the patients harbored the *HER2* codon 655 GG genotype (5 out of 132, 4%) with no association observed with cardiac toxicity (LVEF decrease > 15%, LVEF < 50%), potentially due to the small sample size and low frequency of this patient population, as supported by the authors. A strong association of the *HER2* codon 655 AG with cardiotoxicity was also noted by Gómez Peña et al., 2015 [228], compared to the homozygous allele (95% CI (1.20–12.57), *p* = 0.024) in HER2 breast cancer patients treated with trastuzumab. An even more significant association was observed when menopausal status and use of anthracyclines at baseline were considered (95% (CI 1.43–18.36), *p* = 0.012). Cardiotoxicity was defined as either a decrease in the LVEF by 15% resulting in LVEF < 50%, LVEF decline by less than 45%, LVEF reduction by 15% from baseline, or manifestation of CHF. However, no clear association of the AG polymorphism is provided for each of the specific features used to define cardiac toxicity (e.g., decline in LVEF) in this study. The proposed association of *HER2* codon 655 AG polymorphism with cardiotoxicity is in line with the result of a meta-analysis conducted by the authors in order to increase the sample size and statistical power. The meta-analysis involved three previously published studies, two of which are included in this review [227,229] and one conducted in 2007 [230]. The meta-analysis showed a significant association of the *HER2* codon 655 AG polymorphism with cardiotoxicity as opposed to the homozygous allele (OR = 5.35, 95% CI (2.55–11.73), *p* < 0.0001) whilst the *HER2* codon 655 GG polymorphism had no correlation with cardiotoxicity [228].

The retrospective polymorphism sub-study [229], was conducted including 73 patients of which 52 patients had the homozygous allele, *HER2* codon 655 AA (71%), 18 patients had the heterozygous allele, *HER2* codon 655 AG (25%) and 3 patients had the *HER2* codon 655 GG (4%). A higher risk to develop cardiac toxicity was noted in 33% of the patients with the *HER2* codon 655 AG compared to the 8% of the patients with the *HER2* codon 655 AA (OR = 5.87, 95% CI [1.33–25.82], *p* = 0.02). Similarly to the other studies, patients with the *HER2* codon 655 GG did not experience cardiac toxicity, which was defined as LVEF decreasing by at least 10% with LVEF < 50% or any decrease resulting in LVEF < 45%.

Overall, a common limitation of the four studies is the small sample size, which did not allow for powerful statistical analysis. Hence, larger cohorts are required in order to validate the association of *HER2* codon 655 AG and/or *HER2* codon 655 GG polymorphisms with cardiotoxicity induced by trastuzumab-directed therapies in breast cancer patients. Despite the fact that the association of *HER2* codon 655 AG with cardiotoxicity is supported by the aforementioned studies, no evidence is provided about the molecular mechanisms through which *HER2* codon 655 AG is correlated with the induction of CTRCD.

Stanton et al., 2015 [222], recruited a total of 140 patients in a single-center prospective study of which 29 cases developed cardiotoxicity. Cardiotoxicity was defined as the clinical manifestation of CHF, or a decline in LVEF of 15% resulting in interruption of trastuzumab treatment, LVEF < 55%, or LVEF decline by 10%. In this study, two polymorphisms among a total of 11 SNPs examined, showed to have variation within the ethnically mixed population. The two SNPs included the *HER2* codon 655 AG and *HER2* codon 1170 CG (Pro1170Ala). In this study, no association of the AG polymorphism with cardiotoxicity was noted (*p* = 0.96); however, this was accompanied by no available statistical data nor correlation analysis with each of the features used to define cardiotoxicity. In contrast, 35% of the patients that develop cardiotoxicity (10 out of 29 patients, *p* = 0.04) harbor the *HER2* codon 1170 CC as opposed to the 17.1% of the patients with *HER2* codon 1170 CG in the non-cardiotoxic control group (19 out of 111).

A larger cohort of a three-fold greater number of patients (n = 800) [226], compared to the three aforementioned prospective studies combined (n = 300) [221,227,228], revealed no association of either polymorphism, *HER2* codon 655 AG and *HER2* codon 1170 CC, with cardiotoxicity (*p* > 0.05). This was noted based on both linear and logistic regression models and using the definition of cardiotoxicity as per the previously published studies including the more stringent definition of Gómez Peña et al., 2015 [228]. For the lack of data reproducibility, the authors excluded the reason for genotyping errors, as multiple probes were tested, and allele frequencies were in agreement with published studies. It was supported that the lack of data replication is due to the small sample size of the previously published studies, which demonstrated the association of these variants with cardiotoxicity. In parallel, the authors managed to replicate previously published data on the association of genetic variants in the *ABCB1* (*p* = 0.018) and *CBR3* genes (*p* = 0.004) with chemotherapy-induced cardiotoxicity using doxorubicin.

In conclusion, further studies with a higher number of participants are required in order to validate the role of SNPs mentioned above and achieve adequate statistical power.

##### Other SNPs

Other studies have investigated the association of other variants with CTRCD including the SNP, rs28714259, which was identified using a genome-wide association study (GWAS) and shown to be associated with CHF (*p* = 0.041, OR = 1.9) and decreased in LVEF < 45% (*p* = 0.018, OR = 4.2) in response to adjuvant treatment with paclitaxel, cyclophosphamide and anthracyclines including doxorubicin [231]. The rs28714259 SNP is found in the glucocorticoid receptor (GR) binding site and it was shown to decrease its binding affinity in vitro and in vivo [232]. The SNP was identified in a randomized phase III breast cancer trial ECOG-E5103 (cohort sample size n = 4994, tumor-derived DNA from n = 3431, OR = 2.1; *p* = 9.25 × 10^−6^) and validated in two independent phase III breast cancer trials, E1199 (cohort sample size n = 5052, tumor-derived DNA from n = 2906, OR = 1.9; *p* = 0.04) and BEATRICE (cohort sample size n = 2591, tumor-derived DNA from n = 828, OR = 4.2; *p* = 0.018) [231].

The case group included breast cancer patients with CHF (as defined by the NYHA class III or IV for BEATRICE or by the Common Toxicity Criteria version 2.0 for E1199) or cases of cardiac events (decline in LVEF > 10% to LVEF < 50%). The control groups included patients without CHF or other cardiac events (LVEF < 50% or drop by LVEF ≥ 20% from baseline (E5103) or LVEF < 45% (BEATRICE). Raw LVEF values were not available from the E1199 trial. Association of the rs28714259 with CHF and drop in LVEF < 45% were validated as part of E1199 and BEATRICE trials, respectively [231]. The predictive role of this SNP was also validated by Gvaldin et al., 2021 [233], in a study of 256 patients of which 235 patients had no CV complications and 21 patients experienced cardiac events. It was shown that the rs28714259 SNP significantly increased the risk of cardiotoxicity and the incidence of impaired LV contractility by 3.3-fold (95% CI (1.23–8.75), *p* = 0.002) and 6.6-fold (95% CI (1.92–22.75); *p* = 0.003), respectively. Cardiac events included a reduction in LVEF > 10%, ECG abnormalities, arrhythmia, cardialgia, and pronounced dyspnea.

A recent publication by Wu et al., 2022 [232], revealed insights into the mechanisms through which rs28714259 polymorphism may trigger cardiac failure in response to anthracyclines. It was shown that rs28714259 disrupts the GR-mediated protective signaling pathway against doxorubicin-induced cardiotoxicity and reduction in cardiac contractility, dysregulates cardiac hypertrophy signaling, mitochondrial function, and glucose metabolism, which interferes with cardiomyocyte survival following doxorubicin using human induced pluripotent stem cell-derived cardiomyocytes (iPSC-CMs) in vitro [232].

Two studies investigated the role of the uridine glucuronosyltransferase 2B7 (*UGT2B7*)–161 SNP C to T (C > T) (rs7668258) and the incidence of cardiotoxicity in Chinese breast cancer patients [234,235]. UGT2B7 is a liver enzyme, which mediates drug elimination through glucuronidation. Glucuronidation enhances the water solubility of lipophilic anti-cancer agents and in turn, increases the clearance rate of these drugs and their toxic metabolites from the body. UGT2B7 polymorphisms were shown to differentially regulate drug metabolism and drug-associated toxicity [236]. Both studies [234,235], demonstrated that the patients with UGT2B7-161 CC are at higher risk to experience cardiotoxicity compared to the patients with UGT2B7-161 TT and UGT2B7-161 CT. Specifically, Li et al., 2022 [235], included 50 post-operative HER2 breast cancer patients registered to receive trastuzumab in combination with pertuzumab, without a diagnosis of severe cardiac complications and/or hypertension, thoracic abnormalities or endocrine system diseases. Cardiotoxicity was defined as a drop in LVEF by at least 10% and/or reduction in LVEF < 53%. However, the time points assessed were limited including the baseline and 3 months post-treatment. Li et al., 2019 [234], included a total of 427 post-operative breast cancer patients scheduled to receive epirubicin, cyclophosphamide followed by docetaxel. Concurrent treatment with trastuzumab and docetaxel was administered in patients with HER2-positive breast cancer. Cardiotoxicity was defined as the drop in LVEF by at least 10% from baseline resulting in LVEF < 53% or clinical manifestation of acute coronary artery syndrome, CHF, or arrhythmias. The follow-up period was for one-year post-treatment. Both studies revealed that the UGT2B7-161 T allele is independently associated with low incidences of cardiotoxicity compared to the CC genotype (*p* = 0.004). It was previously shown that breast cancer patients with UGT2B7-161 TT or CT had increased epirubicin clearance rate (median, 134.0 L/h; *p* = 0.002) compared to the patients with UGT2B7-161 CC (median, 103.3 L/h) [236]. It is believed that UGT2B7-161 C>T, which is found on the promoter region of *UGT2B7*, stimulates its transcription and enhances UGT2B7 enzymatic activity. This then triggers glucuronidation of epirubicin and increases its clearance rate from the body, hence protecting cardiac cells from potential epirubicin-induced cardiotoxicity [234,236]. However, both studies [234,235] had several limitations including selection bias as all patients were Chinese from a single center, the small sample size, and the short period of follow-up.

Nakano et al., 2019 [223], conducted GWAS to identify novel SNPs predicting trastuzumab-induced cardiotoxicity in a total of 268 Japanese patients of which 11 cases experienced cardiotoxicity and the remaining 257 controls had no incidences of cardiotoxicity. Cardiotoxicity was defined as a reduction in LVEF < 45% or a drop by 10% from baseline resulting in LVEF < 50%. GWAS revealed a total of 100 SNPs to be associated with cardiotoxicity and which were further validated in a replication study, which included 14 cases with cardiotoxicity compared to 199 controls with no cardiotoxicity. Combined results of both GWAS and the replication study identified five SNPs to be significantly associated with a higher risk of trastuzumab-induced cardiotoxicity. These were the following: rs9316695 (p_combined_ = 6.00 × 10^−6^, OR = 4.46, 95% CI (2.30–8.47)), rs28415722 (p_combined_ = 8.88 × 10^−5^, OR = 5.48, 95% CI (2.21–13.69)), rs7406710 (p_combined_ = 1.07 × 10^−4^, OR = 6.64, 95% CI (2.19–27.01)), rs11932853 (p_combined_ = 1.42 × 10^−4^, OR = 3.20, 95% CI (1.70–6.23)) rs8032978 (p_combined_ = 1.60 × 10^−4^, OR = 5.83, 95% CI (2.30–13.51)). A prediction model developed using the five SNPs showed that the presence of these SNPs could predict trastuzumab-induced cardiotoxicity at baseline (*p* = 7.82 × 10^−15^, OR = 40, 95% CI (15.6–102.3)). A follow-up study by the same authors, showed that the two previously identified SNPs, rs8032978 (p_combined_ = 4.92 × 10^−5^, OR = 3.49) and rs7406710 (p_combined_ = 5.50 × 10^−5^, OR = 3.47) had a stronger association with trastuzumab-induced cardiotoxicity compared to the remaining three SNPs, in a case–control cohort study consisting of both Japanese (6 cases and 206 controls) and Singaporean (22 cases and 178 controls) patients with HER2+ breast cancer treated with trastuzumab [237]. Cardiotoxicity was defined as previously by Nakano et al., 2019 [223]. However, it is worth noting that the strong association was evident only after a combined analysis with the previous study [223]. Otherwise, only similar trends of association to the five SNPs with trastuzumab-induced cardiotoxicity were noted in this study, without significance, potentially due to the small sample size [237]. Larger-scale validation studies need to be conducted to verify the association of the selected SNPs with trastuzumab-induced cardiotoxicity.

#### 3.3.2. MicroRNAs

MicroRNAs are endogenous small single-stranded non-coding RNAs (18–25 nucleotides), implicated in various diseases including CV diseases such as CHF [238], acute myocardial infarction [239,240] as well as metabolic disorders [241] and diabetes [242,243,244]. MicroRNAs can regulate protein expression post-transcriptionally via binding to protein-coding messenger RNA (mRNA) [245]. Several studies have reported the potential of using miRNAs as diagnostic biomarkers, primarily due to their direct association with specific diseases [244,246,247], high sensitivity, and biochemical stability [248,249]. Studies have demonstrated that the levels of circulating miRNAs are correlated with CTRCD [250] (Table S2). A total of 15 prospective studies published during 2013–2023, revealed a total of 445 differentially expressed miRNAs in breast cancer patients experiencing cardiotoxicity compared to patients without cardiotoxicity. Specifically, from the 445 miRNAs, 229 miRNAs were found to be downregulated and 216 were upregulated in relation to cardiotoxicity [89,95,144,251,252,253,254,255,256,257,258,259,260,261,262]. Sánchez-Sánchez et al., 2022 [251] revealed that miR-4732-3p, one of the most promising cardioprotective microRNAs, was downregulated in the blood samples of breast cancer patients following anthracycline treatment (* *p* < 0.05 validation study; ** *p* < 0.01 main study). MiR-4732-3p was the only miRNA identified by three independent regression models (elastic net, Robinson and Smyth exact negative binomial test, and random forest). The prospective observational study included patients with declined cardiac function (n = 10 cases) during year 1 post-treatment with anthracycline-based chemotherapy and patients without cardiac dysfunction (n = 10 controls). The findings were validated by a second cohort (7 cases and 25 controls). Cardiotoxicity was defined as a symptomatic reduction in LVEF by 5%, resulting in LVEF < 55%, or an asymptomatic reduction in LVEF by 10% resulting in LVEF < 55%.

Significant upregulation of the circulating miRNAs, known to be implicated in cardiac remodeling, cardiac dysfunction, and cardiomyocyte apoptosis, was recently demonstrated in response to trastuzumab in combination with taxanes [89]. This is a prospective study of a total of 17 HER2+ breast cancer patients treated with trastuzumab in combination with paclitaxel or docetaxel (with or without carboplatin). A total of 17 healthy subjects were included as the control group. Specifically, miR-1, miR-21, and miR-30e were statistically significantly increased at 3 and 6 months of trastuzumab treatment compared to the baseline and to the healthy controls (*p* < 0.05). MiR-34a and miR-133 showed significant upregulation at 6 months as compared to the control (*p* < 0.05) and the control and baseline (*p* < 0.05), respectively. Upregulated levels of cTnI and cTnT were noted in response to trastuzumab and were strongly correlated with the increased levels of miR-34a (r = 0.3394 cTnI; r = 0.3882 cTnT), miR-21 (r = 0.4036 cTnI; r = 0.4744 cTnT), miR-133 (r = 0.5804 cTnI; r = 0.4242 cTnT), miR-1 (r = 0.3235 cTnI; r = 0.4372 cTnT) and miR-30e (r = 0.3350 cTnI; r = 0.4920 cTnT) with all at >99% CI. The authors concluded that the miRNA panel identified can be a promising screening tool for the early identification of cardiotoxicity induced by trastuzumab-based therapy. Cardiotoxicity was defined as LVEF reduction by ≥5% resulting in LVEF < 55% compared to baseline with symptomatic HF or as an asymptomatic HF with LVEF reduction by ≥10% to LVEF < 55% from baseline. In this study, there was no association between the miRNA panel and LVEF reduction, potentially due to the small sample size and the low incidences of cardiotoxicity. Despite the strong statistically significant data of the study, the authors acknowledge the short follow-up time and the need to evaluate the miRNA panel in the largest cohort with patients at a higher risk of CTRCD.

Upregulation of circulating miRNAs was also noted by Lakhani et al., 2021 [95] in breast cancer patients (n = 17) at 3 and 6 months of anthracycline chemotherapy compared to healthy subjects (n = 17). Specifically, increased levels of miR-423, miR-126, miR-34a, and miR-29a were detected at 6 months as compared to the baseline (*p* < 0.05 or *p* < 0.01) and healthy individuals (*p* < 0.01). MiR-499 levels were increased at 6 months as compared to the control (*p* < 0.01), miR-126 levels were upregulated at 3 months as compared to the control (*p* < 0.01), and baseline (*p* < 0.05) and miR423 levels were increased at month 3 as compared to the control (*p* < 0.01). The increased levels of miRNAs were positively correlated with elevated troponins as follows: cTnI was strongly correlated with miR-29a, miR34a, and miR-126 (r = 0.3052; r = 0.3163; r = 0.6164, respectively). Significant correlation was observed between the hscTnT and miR-126, miR-423 and miR-499 (r = 0.4886; r = 0.3638, r = 0.3959, respectively). In contrast to the previous study by the same research group [89], no significant correlation was noted between troponin T and miR-34a nor miR-29a.

In addition, the levels of miR-3135b were significantly upregulated in breast cancer patients with chemotherapy-induced cardiotoxicity (n = 33) (*p* = 0.0001) compared to the patients without cardiotoxicity (n = 37) [252]. The upregulated levels of miR-3135b were strongly correlated with changes in LVEF (r = 0.5, *p* = 0.0001). In addition, patients with HF had significantly higher levels of miR-3135b (*p* < 0.05) compared to the control group. Patients were treated with anthracycline-based regimens followed by treatment with paclitaxel (67.4%) or docetaxel (26.1%) and cardiotoxicity was defined as a reduction in LVEF < 50%. In this study, the levels of miR3135b prior to each treatment cycle were not evaluated and hence further studies are needed to assess multiple time points during treatment.

The levels of miR-130a, known to be implicated in the regulation of cardiac pathology, were progressively increased in HER2+ breast cancer patients with (*p* < 0.001) or without cardiotoxicity (*p* < 0.001) during adjuvant treatment with epirubicin/cyclophosphamide followed by docetaxel and trastuzumab [144]. However, in patients with cardiotoxicity, the magnitude of increase in the miR-130a levels was greater in the cardiotoxicity patients at all time points (all *p* < 0.05) assessed during treatment as revealed by a two-group comparison analysis. Importantly, baseline levels of miR-130a were negatively and positively correlated with changes in LVEF and cTnI (*p* = 0.038, Spearman r = −0.245; *p* < 0.001; Spearman r = 0.414, respectively), respectively. However, no correlation was evident with NT-proBNP (*p* = 0.112, Spearman r = 0.019). Baseline levels of miR-130a (AUC = 0.783; 95% CI (0.647–0.920)) distinguish patients who experience cardiotoxicity from patients without cardiotoxicity. In this study, cardiotoxicity was defined by a reduction in LVEF by ≥10% and LVEF < 53% or HF defined as LVEF < 40% or BNP > 35 ng/L plus NT-proBNP >1 25 ng/L or acute coronary artery syndrome or severe arrhythmia. Despite the evidence provided, the molecular mechanism through which miR-130a may trigger cardiac damage and CTRCD, remains unknown and requires in vitro/in vivo experiments. Limitations of the study include the small sample size and the potential bias that may have been introduced due to variability in the LVEF measurements, which may have been different according to the sonographer and the heart rate.

Evidence demonstrated that HER2+ breast cancer patients with low expression of miR-222-3p showed complete response (31 out of 65 patients, 4.69%) (OR = 0.258, 95% CI (0.070–0.958, *p* = 0.043)), disease-free survival (HR = 5.778, 95% CI (1.196–27.906), *p* = 0.029) and overall survival (*p* = 0.0037) [253]. Expression of serum miR-222-3p could predict trastuzumab-induced cardiotoxicity (OR = 0.410, 95% CI (0.175-0.962), *p* = 0.040). In this study, a total of sixty-five breast cancer patients were enrolled, derived from two neoadjuvant clinical trials (SHPD001 and SHPD002). All patients received neoadjuvant paclitaxel and cisplatin followed by trastuzumab.

Interestingly, downregulation of the levels of miRNA-548 was noted in the PBMCs of patients with dilated cardiomyopathy (DCM) (NYHA class II/III) (n = 44) but no changes were noted in metastatic breast cancer patients with normal cardiac function or patients with coronary artery disease (n = 10). This suggests that changes in miRNA548 expression in PBMCs may be selective to DCM and hence it was suggested as a promising indicator of early cardiac dysfunction [255].

#### 3.3.3. Myeloperoxidase (MPO)

Myeloperoxidase (MPO) is a myeloid-lineage restricted enzyme with bactericidal properties, found in the azurophilic granules of neutrophils [263], the main source of MPO [264]. MPO is a component of the neutrophil extracellular traps (NETs) [265]. NETs are produced by neutrophils and are composed of DNA structures, released as a result of decondensed chromatin, histone proteins, and more than 30 granule proteins including components with antibacterial activity such as neutrophil elastase, MPO, cathepsin G, and peptidoglycan-binding proteins [163,266,267]. Evidence implicated NETs and NETs-related factors such as dsDNA, MPO/DNA complexes with myocardial infarction, and serious cardiac events [268]. Upon MPO activation, MPO by-products are extremely oxidizing agents such as the hypochlorous acid, HCIO- resulting in molecular damage and can be implicated in oxidative stress resulting in cellular damage [163,269,270]. Subsequent studies detected elevated concentrations of neutrophil-specific genes including MPO in patients who experienced doxorubicin-induced cardiotoxicity [95,105,271]. Consequently, the utility of using MPO as a predictive biomarker of CTRCD was investigated in several clinical studies [95,130,271,272] (Table S2).

MPO was one of the plasma biomarkers assessed as a potential novel indicator of doxorubicin-induced cardiotoxicity in breast cancer patients in a study of 17 patients with triple-negative breast cancer patients and 17 healthy individuals [95]. It was shown that MPO was significantly elevated at 3 and at 6 months in patients receiving doxorubicin as compared to the healthy control group (*p* < 0.01) and baseline (*p* < 0.01). Cardiotoxicity was detected in a total of four patients (23.5%) at 6 months of treatment showing an LVEF reduction of 8.1%, 7%, 9.3%, and 11.5%, respectively. Cardiotoxicity was defined by either LVEF reduction of ≥5% resulting in LVEF < 55% with clinical manifestation of CHF or by an asymptomatic drop in LVEF by ≥10% resulting in LVEF < 55% from baseline. A significant positive correlation was noted between elevated levels of cTnI and cTnT with higher levels of MPO (r = 0.3078 and r = 0.3240, respectively). These findings suggest that MPO can be used as a promising biomarker for the detection of the early onset of anthracycline-related cardiac dysfunction even before detected by echocardiographic assessment. However, the small sample size in this study did not allow for the prediction of cardiotoxicity in a larger population group.

Elevated circulating levels of MPO were detected in breast cancer patients treated with anthracyclines (n = 192) as part of the CECCY trial with 1.3- and 1.5-fold increase at 3 months (17.7 ng/mL (11.1, 31.1)) and 6 months (19.2 ng/mL (11.1, 37.8)) after initiation of treatment, respectively, compared to the baseline (13.2 ng/mL (7.9, 24.8)). However, the levels of MPO did not differentiate (*p* = 0.85) between the patients with decreased LVEF by ≥10% (14.1 ng/mL (10.4, 25.5), n = 26) compared to the patients without a decrease in LVEF (18.1 ng/mL (12.3, 39.1)), n = 148) at 3 months and 6 months (16.3 ng/mL (10.3, 35.6) and 20.5 ng/mL (10.5, 37.8), respectively), after initiation of anthracycline-based chemotherapy. However, higher levels of baseline MPO above the median were associated with elevated levels of cTnI (*p* = 0.041) during treatment (6, 9, and 12 months), indicative of myocardial injury. The authors concluded that higher MPO levels prior to chemotherapy can be used to identify patients with a higher probability to benefit from the cardioprotective effects of carvedilol (*p* < 0.001) [271]. In this study, cardiotoxicity was defined as a drop in LVEF by ≥10% at any point until 6 months when chemotherapy is completed. It is worth noting that since there was no established cut-off value for MPO, similar cut-off values were applied by the authors as described previously [273]. In addition, the low incidences of cardiotoxicity observed in the CECCY trial may have underpowered the ability to detect a potential association between MPO and the development of anthracycline-related cardiac dysfunction.

MPO levels were significantly elevated in HER2+ breast cancer patients by 3 months of treatment and were significantly associated with cardiotoxicity during the entire treatment course of doxorubicin/trastuzumab with HR = 1.37 (95% CI (1.11–1.69); *p* = 0.02) [98]. The authors showed that persistent elevated MPO levels after the 3 months of treatment can be used to predict patients at high risk of cardiotoxicity in response to doxorubicin/anthracycline treatment. A total of 78 patients with breast cancer were included in this study and 23 patients developed 39 cardiac events over the treatment course. Cardiotoxicity was defined in accordance with the Cardiac Review and Evaluation Committee definition [67]. Similarly, another study showed higher serum levels of MPO at 3 months of treatment compared to the baseline (*p* < 0.05) in a multicenter cohort of 78 HER2+ breast cancer patients treated with adjuvant doxorubicin, taxanes, and trastuzumab [105]. Greater predicted risk of cardiotoxicity was significantly associated with an increase in interval change in the levels of MPO (HR = 1.36 per SD; 95% CI (1.04–1.79); *p* = 0.03). In particular, the risk of cardiotoxicity by month 15 was 36.1% in patients with greater changes in the levels of MPO (ΔMPO > 422.6 pmol/L). Elevated changes in both cTnI and ΜPO increased the probability of cardiotoxicity to 46.5%. Cardiotoxicity was defined in accordance with the Cardiac Review and Evaluation Committee as previously described [67].

A total of 51 participants with ER+PR+HER2- or TNBC breast cancer were treated with neoadjuvant (n = 36) or adjuvant (n = 15) doxorubicin in combination with cyclophosphamide in the study by Todorova et al., 2020 [163]. Abnormal LVEF was defined as a reduction in LVEF by >10% or LVEF < 50%. Among 51 patients, 21 experienced asymptomatic reduction in LVEF > 10% compared to baseline and the remaining 30 patients had an LVEF decline of ≤10%. Baseline MPO levels were significantly higher in patients with abnormal LVEF (group 1) with a mean value ( ± SD) of 169 ± 50.7 ng/mL compared to the patients with normal LVEF (group 2) with a mean ± SD of 132.6 ± 45.6 ng/mL (*p* = 0.02 model 1; *p* = 0.01 model 2). MPO levels remained increased in group 1 (mean ± SD (269.6 ± 112.5 ng/mL)) compared to group 2 (mean ± SD (174.5 ± 76.0)) after the first cycle of chemotherapy (*p* = 0.007 model 1; *p* = 0.04 model 2) [163]. Overall, evidence from this study further supports the utility of MPO as a promising predictor of cancer therapy-induced cardiotoxicity, even at a baseline level. Gullo et al., 2019 [93], showed that significantly increased serum MPO levels were detected in HER2-negative breast cancer patients at 3 months (mean ± SD (1.627 ± 4.73), *p* < 0.05) of adjuvant treatment with docetaxel and cyclophosphamide in combination with bevacizumab compared to the baseline (mean ± SD (0.95 ± 1.47)). However, the elevated levels of MPO were not differentiated between the cardiotoxic (n = 12) versus non-cardiotoxic (n = 50) patients at either the baseline or at 3 months of treatment (difference in means 0.57, 95% CI (−0.19, 1.34), *p* = 0.1637) [93].

#### 3.3.4. Galectin-3 (Gal-3)

Galectin-3 (Gal-3) is a β-galactoside-binding protein, a member of the lectin family, implicated in various pathophysiological processes including fibrosis, inflammation, oxidative stress [274] and is known to induce cardiac fibroblast proliferation and collagen production and deposition [275]. During CHF, activated myocardial macrophages and cardiac fibroblasts release Gal-3 [275,276]. Gal-3, as a marker of cardiac fibrosis, is considered to be a promising predictor of the onset of CHF and related mortality in patients [277].

Recent studies investigated Gal-3 as a potential diagnostic biomarker for cancer therapy-induced cardiac dysfunction in breast cancer patients [92,136,149,271,278,279] (Table S2). A total of seven studies were included in this review, with five studies revealing changes in the circulating levels of Gal-3 in response to treatment with cardiotoxic breast cancer therapies [92,105,149,271,279]. The five studies included a total of 505 breast cancer patients with Gulati et al., 2017 [92], showing significantly increased levels of Gal-3 in patients treated with adjuvant anthracycline epirubicin combined with 5-FU and cyclophosphamide (median value 13.4 ng/mL (11.2, 16.0); *p* < 0.001) compared to baseline (median value 12.1 ng/mL (10.4, 14.0)). However, there was no association between the elevated levels of Gal-3 and the incidence of cardiotoxicity, which was defined as previously described [67]. Similarly to Gal-3, no association with LV dysfunction was noted with either of the other biomarkers assessed (cTnI, cTnT, BNP, NT-proBNP, CRP) as identified using multivariable linear regression analysis, which corrected for age, BMI, blood pressure, anthracycline dose and treatment with cardioprotective agents. The lack of association may be due to the small number of patients included in the study and the fact that only one patient showed a reduction in LVEF (from 62.7% to 51%) without HF and hence met the criteria for cardiotoxicity. Additional limitations in the study were the lack of long-term follow-up after adjuvant treatment.

In the study by Bulten et al., 2015 [149], increased levels of Gal-3 were significantly (*p* < 0.05) associated with delayed planar whole-heart (WH) heart/mediastinum (H/M) ratio measured by ^123^I-*metα*iodobenzylguanidine (^123^I-*m*IBG) scintigraphy. This study included 59 breast cancer survivors, 1 year after treatment with anthracyclines (docetaxel, doxorubicin, and cyclophosphamide). In another study by Van Boxtel et al., 2015 [279], abnormal levels of Gal-3 were detected in 7.3% of breast cancer patients (4 out of 55 patients) after treatment with anthracyclines. Two of these patients had diminished LVEF or elevated NT-proBNP, respectively. However, due to the small sample size, the role of Gal-3 as a predictor of early onset of cardiotoxicity could not be sufficiently addressed. In addition, increased Gal-3 levels were noted in patients during chemotherapy with a median difference of 0.5 (−1.5 to 2.2) between baseline and 3 months, which was, however, non-statistically significant (*p* = 0.14) [105]. The elevated levels of Gal-3 were not significantly associated with the risk of cardiotoxicity (HR = 1.33, 95% CI (0.86–2.05); *p* = 0.195). Cardiotoxicity was defined by a decline in LVEV of ≥ 5% resulting in LVEF < 55% with clinical manifestation of HF or by an asymptomatic decline in LVEF by ≥10% resulting in <55% [105].

A post hoc analysis by Wanderley et al., 2022 [271], of breast cancer patients included in the CECCY trial, revealed that Gal-3 levels remained unchanged among the patients with dropped LVEF of least 10% (n = 26; 10.4 ng/mL (8.5, 12.6)) compared to the patients with no change in LVEF (n = 148; 10.3 ng/mL (7.6, 12.5)) at 6 months after initiation of anthracycline treatment (*p* = 0.85). However, elevated levels of Gal-3 were noted in response to anthracycline treatment with approximately 2- and 1.6-fold increases at 3 months (12.3 ng/mL (9.8, 16.0)) and 6 months (10.3 ng/mL (8.2, 13.1)), respectively, compared to the baseline (6.3 ng/mL (5.2, 9.6)) [271]. It is important to note that since there are no established cut-off values for Gal-3, the authors followed a similar division performed in previous studies [98,105].

In the remaining two studies, a total of 658 breast cancer patients were included showing no changes in the levels of Gal-3 in patients receiving breast cancer therapies including anthracyclines and/or trastuzumab nor an association with the development of CTRCD in these patients [98,136]. Specifically, no significant differentiation in the levels of Gal-3 was noted in a total of 580 breast cancer survivors treated with anthracycline-based chemotherapy with a mean (SD) of 15.8 ng/L (7.5) compared to the control group of total 580 patients, who have not received anthracycline-driven therapy with a mean (SD) of 16.1 ng/L (7.8) [136].

The second study by Putt et al., 2015 [98], is a multicenter cohort of a total of 78 HER2+ breast cancer patients treated with adjuvant doxorubicin followed by taxanes and trastuzumab. Cardiotoxicity was defined as a reduction in LVEF by ≥5% to LVEF < 55% with HF or as an asymptomatic drop in LVEF of ≥10% to LVEF < 55 [67]. A total of 23 patients experienced a total of 39 cardiac events during month 15 of the study. There were no significantly increased concentrations of Gal-3 at month 3 compared to the baseline in response to the treatment, as opposed to the other biomarkers assessed (e.g., MPO, PIGF, GDF-15, hs-cTnI). However, a significant association of Gal-3 with the risk of cardiotoxicity was noted at a subsequent visit (HR = 1.60, 95% CI (1.12–2.28); *p* = 0.04), which is, however, not specified.

#### 3.3.5. Matrix Metalloproteinases (MMPs)

Matrix metalloproteinases (MMPs) are proteolytic endopeptidases implicated in extracellular matrix remodeling, secreted by stroma and cancer cells [280,281]. Clinical and preclinical studies have demonstrated the role MMPs as biomarkers of myocardial fibrosis and cardiomyopathies [282,283]. A prospective, observational clinical study by Grakova et al., 2022 [284], showed elevated serum levels of MMP2 and *MMP9* by 8% (*p* = 0.017) and 18.4% (*p* < 0.001), respectively, in patients, who developed anthracycline-induced cardiotoxicity (AIC) (group 1) compared to patients that did not (group 2). The higher levels of MMP2 were associated with changes in LVEF (r = -674; *p* < 0.05), end-diastolic and end-systolic dimension (r = 0.296 and r = 0.399, respectively), NT-proBNP (*p* = 0.568), and MMP9 (r = 0.634) in patients with AIC. Importantly, both MMP2 and MMP9 could predict AIC based on ROC analysis, in contrast to NT-proBNP and echocardiographic parameters, which did not seem to have a prognostic role. In contrast, in group 2, MMP2 and MMP9 were significantly decreased at 24 months. Interestingly, gene polymorphism (C/C genotype) in MMP2 (rs243865) was associated with the progression of anthracycline-induced CHF with a significant decrease in LVEF by 13.2% (from 50% (47,52) to 43% (35; 49)) and increased in end-systolic and end-diastolic dimension by 7.7% (*p* < 0.001) and 4.0% (*p* < 0.001), respectively. Similarly, the C/C genotype of MMP9 (rs3918242) was also related to worsening HF with declined LVEF and increased LV dimensions also accompanied by a significant 15.7% decrease in the levels of NT-proBNP (*p* = 0.052). The authors concluded that the elevated levels of MMP2 and MMP9 in response to doxorubicin and the gene polymorphism identified to be correlated with AIC could be potentially used to assess the risk of AIC in breast cancer patients and indicate the use of prophylactic therapies at the later stages of AIC. This study has focused on the late AIC during the 24-month follow-up period using a relatively small sample size. Further studies are needed to further evaluate the predictive role of MMPs for early and late stages of AIC in breast cancer patients.

Evidence obtained by Kirkham et al., 2022 [285], in a prospective study of 94 HER2-positive breast cancer patients, showed statistically significant higher levels of MMP2 in patients treated with 5-FU, epirubicin, cyclophosphamide (FEC) prior to trastuzumab treatment (234 ± 37 vs. 191 ± 45 ng/mL, *p* < 0.001, respectively). MMP2 levels increased in all treatment groups at post-cycle 4, then returned to baseline levels at post-cycle 17. In this study, the association between MMP2 elevation and LV impairment as an indicator of cardiotoxicity was not evaluated due to the low incidences of LV dysfunction and the small sample size. However, elevated septal extracellular volume (ECV) fraction at post-cycle 4 (24.3 ± 3.4%, *p* = 0.004) compared to baseline (22.9 ± 3.3%), was used to identify early myocardial edema in all patients as also supported by the subsequently elevated levels of GDF-15, an indicator of myocardial inflammation.

### 3.4. Other Biomarkers

Potential biomarkers of cardiotoxicity either recently reported or currently under investigation are presented in Table 1.

#### 3.4.1. Inflammatory Biomarkers

In a prospective study by Alves et al., 2022 [125], of a total of 64 breast cancer patients, the plasma levels of inflammatory cytokines including interleukin (IL)-1β, IL-6, IL-10 and tumor necrosis factor (TNF) were assessed in response to doxorubicin therapy. Amongst the inflammatory cytokines assessed, only IL-10 levels were significantly increased in patients, who experienced cardiotoxicity (n = 22) at 7d (*p* = 0.006) and at 12 months (*p* = 0.046) post-treatment compared to the non-cardiotoxicity group (n = 42). The levels of IL-10 were significantly correlated with the levels of NT-proBNP at baseline (r = 0.427, *p* = 0.048) and at 12 months post-treatment (r = 0.740, *p* = 0.004) as well as with IL-1β at 7 days post-treatment (r = 0.488, *p* = 0.021).

Another prospective study showed elevation of the levels of pro-inflammatory markers, free iron (Fe), tumor necrosis factor alpha (TNF-α) and homocysteine were elevated in breast cancer patients treated with doxorubicin (DOX; n = 33), paclitaxel (PTX; n = 35) or trastuzumab (TZ; n = 52) compared to the healthy controls (n = 50). These levels were accompanied by elevated levels of CK-MB and hs-CRP, indicating that the proinflammatory markers may potentially be used as promising predictive indicators of cancer therapy-induced cardiac injury [286]. Tromp et al., 2020 [153], identified a distinct biomarker profile in breast cancer survivors (n = 342), who experienced cardiac dysfunction even a decade post-treatment with chemotherapy compared to the control group (n = 346). Amongst the elevated biomarkers, a total of 11 genes were significantly associated with a reduction in LVEF in breast cancer survivors.

Distinct chemokine and immunological signatures were identified in a pilot biomarker study by Yu et al., 2018 [287], in breast cancer patients at baseline and at the early stages of treatment with doxorubicin plus cyclophosphamide.

Differential expression of the biomarkers assessed, including MMPs, chemokines, and cardiac markers, was observed depending on the cardiotoxicity state of each of three patient groups: cardiotoxicity group (n = 5), sub-cardiotoxicity (intermediate) (n = 5) and non-cardiotoxicity group (normal) (n = 17) as detailed in Table 1. It was concluded that the differences in chemokine abundance and the distinct immunological profiles in breast cancer patients may be useful predictors of DOX-induced cardiotoxicity. It is worth noting that in this study, there were no statistically significant differences in the age among the groups; however, expanded studies are needed to investigate the association of other potentially contributing factors such as pre-treatment with other anti-cancer agents, cancer type, and disease stage, which were not investigated [287].

Todorova et al., 2020 [163], revealed the association of a variety of inflammatory, endothelial, and coagulation-related factors as predictive indicators of DOX-induced cardiotoxicity at baseline (T0) and after treatment (T1). A statistically significant increase was noted for NET nucleosomes, thrombin–antithrombin complex (TAT), thrombomodulin (TM), and CRP in breast cancer patients with reduced LVEF > 10% (*p* < 0.05) compared to the patients with normal LVEF in response to DOX-based chemotherapy.

#### 3.4.2. Neutrophil-to-lymphocyte Ratio (NLR)

Recently, novel studies have focused on investigating whether elevated neutrophil-to-lymphocyte ratio (NLR) may have a predictive role in the onset and development of cardiotoxicity in breast cancer patients treated with anthracyclines. Baruch et al., 2023 [288], were the first to evaluate the role of NLR in predicting anthracycline-induced cardiotoxicity using LV GLS and revealed that patients with NLR ≥ 2.58, which was set as the cut-off value, had twice as high risk for LV GLS reduction by ≥10% (50% vs. 20%, *p* = 0.009) in response to anthracycline-based chemotherapy. It is interesting to note that the change in the NLR ratio was attributed to the lower levels of absolute lymphocyte counts during treatment, T2, (1.1 [0.8–1.5]; *p* < 0.001) compared to baseline, T1 (1.8 [1.5–2.3]). The authors have also shown that high NLR value was not the only independent predictor, since trastuzumab treatment could also predict LV GLS reduction as demonstrated by two multivariate binary regression models (OR = 4.90; 95% CI (1.33–18.00); *p* = 0.02 and OR = 4.01; 95% CI (1.09–14.7); *p* = 0.04, respectively).

A recent sub-study by Ryu et al., 2021 [289], from a prospective, randomized phase III clinical trial of seven centers, was conducted with 123 HER2-negative breast cancer patients and identified differences in the metabolic and immunologic profiles as well as NLRs in patients treated with neoadjuvant chemotherapy (NCT) or neoadjuvant endocrine therapy (NET). Patients were treated with goserelin acetate and tamoxifen or adriamycin plus cyclophosphamide, followed by docetaxel (Table 1). The study demonstrated that the NLR was elevated by 1.7-fold (*p* < 0.01) after NCT followed by a decrease of 2.2-fold after 3 years. NLR was significantly decreased by 1.4-fold following NET (*p* < 0.05) and increased by 1.12-fold after 3 years (Table 1).

#### 3.4.3. Metabolism

Multimodal therapeutic strategies for breast cancer may induce prolonged alterations in the metabolic profile of patients, which may in turn increase susceptibility to future CV diseases [289].

Ryu et al., 2021 [289], demonstrated that neoadjuvant treatment with adriamycin plus cyclophosphamide followed by docetaxel, induced a significant increase in total cholesterol and fasting glucose, compared to baseline. No significant changes were noted in response to neoadjuvant endocrine therapy that included treatment with goserelin and tamoxifen. However, the study only included a small number of patients.

Giskeødegård et al., 2022 [290], detected significantly increased triglyceride levels (VLDL-2 and -3) in patients receiving radiotherapy (RT) with or without endocrine treatment (Group 4 n = 68 and Group 5 n = 78, respectively). Statistically significant reduction in VLDL-4 triglyceride was noted in patients treated with RT and chemotherapy (Group 2 n = 22) or chemotherapy plus trastuzumab with/without endocrine therapy (Group 3 n = 35). All groups demonstrated statistically lower levels of VLDL-5 triglyceride levels. Importantly, non-survivors seemed to have lower levels of total cholesterol, LDL, and HD compared to the patients who survived 10 years after enrolment in the study. Despite the distinct lipoprotein profiles amongst the treatment groups, the association of these profiles with the risk of CTRCD remains to be fully explored.

Another study by Finkelman et al., 2017 [291] investigated the association between arginine–nitric oxide (NO) metabolites and the development of CTRCD. Statistically significant (*p* < 0.05) higher levels of asymmetric dimethylarginine (ADMA) were noted in the first and second months of treatment with DOX-based chemotherapy, whilst arginine and citrulline levels were statistically significantly decreased.

**Table 1:** Other Biomarkers.

Biomarker	Subjects	Treatment	Time Points	Clinical Outcome	Definition of Cardiotoxicity	References
IL-10, IL-1β, IL-6, TNF	n = 22 cases n = 42 control	Doxorubicin	- Before chemotherapy (T0) - Up to 7 days after the last infusion (T1) and - 12 months after the last infusion (T2)	- Elevated levels of IL-10 in the cardiotoxicity group at T1 (*p* = 0.006) and T2 (*p* = 0.046) - Correlation between IL-10 and NT-proBNP at T0 (*p* = 0.048) and T2 (*p* = 0.004)	LVEF < 50% or declined LVEF > 10% resulting in LVEF < 50% compared to the baseline and/or troponin and NT-proBNP increased by 20% at T1 or T2 compared to T0	Alves et al., 2022 [125]
TNF-α, Homocysteine levels, Free iron	n = 33 doxorubicin group; n = 35 paclitaxel group; n = 52 trastuzumab group; n = 50 healthy individuals	Doxorubicin, Paclitaxel and trastuzumab	- After the first cycle of chemotherapy for the doxorubicin group and paclitaxel group - After the eighth cycle of trastuzumab treatment	- Elevated levels of TNF-α in the DOX group: 42.31 ± 17.96 pg/mL (*p* = 0.01); PTX group: 38.27 ± 9.12 pg/mL (*p* = 0.023); TZ group: 89.6 ± 12.11 pg/mL (*p* = 0.032); compared to the control group: 9.47 ± 1.56 pg/mL - Elevated levels of homocysteine in the DOX group: 9.11 ± 0.83 μmol/L (*p* = 0.021); PTX group: 10.95 ± 0.86 μmol/L (*p* = 0.005) TZ group: 9.95 ± 1.15 μmol/L (*p* = 0.0396) compared to the control group: 7.80 ± 0.397μmol/L - Elevated levels of free iron in the DOX group: 138.8 ± 18.6 ug/dL (*p* = 0.0193); PTX group: 113 ± 18.6 ug/dL (*p* = 0.045) TZ group: 120.5 ± 4.64 ug/dL (*p* = 0.0058)	Use total CK, CK-MB, and hs-CRP for evaluating cardiac toxicity - Augmented levels of CK-MB found in the TZ group (*p* < 0.001) and in PTX group (*p* = 0.0361) - Increased levels of hs-CRP in the DOX group: 4.80 ± 1.23 mg/dL (*p* = 0.0005); PTX group: 7.12 ± 1.87 mg/dL (*p* = 0.0006); TZ group: 3.12 ± 0.68 mg/dL (*p* = 0.095) compared to the control group: 0.66 ± 0.18 mg/dL - No changes in total CK in the DOX group:51.65 ± 7.73 U/L; PTX group: 63.24 ± 8.6 U/L; TZ group: 81.65 ± 6.4 U/L compared to the control group: 68.33 ± 6.5 U/L	Micheletti et al., 2021 [286]
92 CV-related proteins in plasma samples	n = 342 survivors (≥5 years since diagnosis and cancer-free since treatment) n = 346 controls (no cancer diagnosis)	Anthracyclines, trastuzumab. Anti-hormonal therapy	BC survivors at ≥5 years since diagnosis	Significant upregulation (*p* < 0.05) of the genes: *TNFSD13B*, *GAL4*, *MCP1*, *KLK6*, *FABP4*, *GDF15*, *SCGB3A2*, *RARRES2*, *CXCL16*, *PI3*, *IGFBP7*, *CNTN1*, *TIMP4*, *OPN*, *PCSK9*, *PLC*, *CTSZ*, *GAL3*, *TFPI* in the BC survivors compared to the healthy individuals. Correlation of elevated levels of *TNFSF13B (p* = 0.02), *FABP4* (*p* = 0.033), *MCP1* (*p* = 0.011), *RARRES2* (*p* = 0.017), *GDF15* (*p* = 0.002), *CXCL16* (*p* = 0.019), *PI3* (*p* = 0.041), *IGFBP7* (*p* = 0.026), *PCSK9* (*p* = 0.041)*, OPN* (*p* = 0.04)*, PLC* (*p* = 0.011) with lower LVEF in the BC survivors.	ECHO assessment of LVEF reduction	Tromp et al., 2020 [153]
40 chemokines 9 matrix MMPs 33 cardiac markers using multiplex immunoassays in plasma samples and cTnT using the fourth-generation assay	n = 17 normal group n = 5 intermediate group (average LVEF reduction of 6.4%) n = 5 cardiotoxicity (average LVEF reduction 13.2%)	Doxorubicin and cyclophosphamide	- Before Dox (T0) - After the first cycle (T1) - After the second cycle (T2)	Baseline Reduced protein levels in intermediate vs. normal: CXCL1 (196 ± 55 vs. 340 ± 203 pg/mL, *p* = 0.022); CCL3 (10 ± 1.5 versus 17 ± 19 pg/mL. *p* = 0.027); GDF15 (0.66 ± 0.13 versus 1.01 ± 0.48 ng/mL, *p* = 0.027); Haptoglobin (669 ± 237 versus 1181 ± 741 μg/mL, *p* = 0.031) Differential protein abundance in abnormal vs. normal: CCL23 (357 ± 89 versus 201 ± 168 pg/mL, *p* = 0.003); CCL27 (985 ± 239 versus 643 ± 240 pg/mL, *p* = 0.008); CXCL6 (28 ± 11 versus 74 ± 48 pg/mL, *p* = 0.013); sICAM-1 (120 ± 29 vs. 222 ± 98 ng/mL, *p* = 0.003) After Dox (T1) Reduced protein levels in intermediate vs. normal: IL-I6 (315 ± 62 pg/mL, *p* = 0.019) FABP3 (977 ± 137 pg/mL, *p* = 0.011) Myoglobin (26 ± 5 ng/mL, *p* = 0.049) Differential protein abundance in abnormal vs. normal: Increased levels of: CCL23 (422 ± 123 pg/mL, *p* = 0.010) Decreased levels of: CXCL5 (485 ± 54 pg/mL, *p* = 0.002) CCL26 (24 ± 3 pg/mL, *p* = 0.011) CXCL6 (25 ± 9 pg/mL, *p* = 0.004) GM-CSF (15 ± 4 pg/mL, *p* = 0.019) CXCL1 (206 ± 38 pg/mL, *p* = 0.003) IFN-γ (37 ± 5 pg/mL, *p* = 0.012) IL-2 (15 ± 3 pg/mL, *p* = 0.036) IL-8 (10 ± 1 pg/mL, *p* = 0.001) CXCL11 (39 ± 24 pg/mL, *p* = 0.047) CXCL9 (136 ± 21 pg/mL, *p* = 0.006) >CCL17 (60 ± 35 pg/mL, *p* = 0.033) CCL25 (416 ± 61 pg/mL, *p* = 0.023) After Dox (T2) Reduced protein levels in intermediate vs. normal: Myoglobin (24 ± 5 ng/mL, *p* = 0.010) CCL23 (98 ± 65 pg/mL, *p* = 0.020) Elevated protein levels in abnormal vs. normal: MIF (4.0 ± 2.4 ng/mL, *p* = 0.031) CCL23 (442 ± 83 pg/mL, *p* = 0.041).	**Abnormal** (i.e., cardiotoxicity): asymptomatic reduction in LVEF > 10% or LVEF < 50% or reduced LVEF > 5% to LVEF < 55% with HF **Intermediate stage** (sub-cardiotoxicity): Reduced LVEF of 5–10% Subclinical cardiotoxicity: reduced LVEF > 4% or >5% **Normal**: Reduced LVEF of <5%	Yu et al., 2018 [287]
NLR and LV GLS	n = 74 received treatment n = 44 with NLR ≥ 2.58 at T2	All patients received Dox, cyclophosphamide, paclitaxel (in most cases) HER2-positive patients were treated with trastuzumab and then pertuzumab	Blood samples were collected at baseline (T1), during dox (T2) ECHO assessment at baseline (T1) and at the end of dox treatment (T3)	Patients with NLR ≥ 2.58 (T2) showed significant reduction in LV GLS at T3 from baseline compared to patients with NLR < 2.58 (10.6 ± 9.6 vs. 6.1 ± 6.9, *p* = 0.02). Patients with NLR ≥ 2.58 (T2) had twice as higher risk for LV GLS reduction ≥ 10% (50%, *p* = 0.009) as compared to patients with NLR < 2.58 (20%). Baseline NLR (T1) could not significantly predict future LV GLS deterioration after the end of treatment (ROC curve analysis and binary logistic regression) (AUC: 0.59; 95% CI (0.44, 0.75); *p* = 0.24 and OR: 1.07; 95% CI (0.81, 1.4); *p* = 0.65, respectively). No statistically significant change was documented in the absolute neutrophil counts at T2 (4.0 (2.2, 5.6); *p* = 0.90) compared to T1 (4.1 (2.8, 5.2)). The majority of HER2-positive breast cancer patients treated with trastuzumab (11 out of 44, 25%) developed NLR ≥ 2.58.	Significant reduction in LV GLS by ≥10% from baseline	Baruch et al., 2023 [288]
CRP TM, TAT, MPO, vWF, *p*-selectin, nucleosomes, dsRNA	n = 21 with asymptomatic decreased LVEF > 10% n = 30 with LVEF ≤ 10%	DOX and cyclophosphamide	Baseline (T0) and after 1 cycle of DOX chemotherapy (T1)	Elevation in the levels of nucleosomes (mean ± SD 144.3 ± 78.9 vs. 113.4 ± 73.3 AU), TM (4.0 ± 1.1 vs. 3.6 ± 0.9 pg/mL) , vWF (5.4 ± 2.4 vs. 4.9 ± 1.7 mIU/mL), TAT complex (14.3 ± 5.7 vs. 12.4 ± 4.6 ng/mL), dsDNA (135.1 ± 39.2 vs. 131.2 ± 26.9 ng/mL), *p*-selectin (0.104 ± 0.07 vs. 0.099 ± 0.08 ug/mL) and CRP (6.8 ± 2.0 vs. 5.5 ± 2.5 mg/L) in the cardiotoxicity versus non-cardiotoxicity group.	Decline in LVEF > 10% or LVEF < 50% compared to baseline	Todorova et al., 2020 [163]
Metabolic profile (BMI, TC, fasting glucose)	n = 64 NCT n = 59 NET	Goserelin acetate with tamoxifen or adriamycin and cyclophosphamide followed by docetaxel	Baseline and after 24 weeks of treatment and 3 years after the initial clinical visit	Patients with hypertension were higher in the NET group compared to the NCT group. BMI significantly changed over the 3 years of follow-up in patients treated with NCT (22.84 kg/m^2^ at baseline (95% CI 21.94–23.74) and 23.87 kg/m^2^ (95% CI 23.06-24.68) after NCT). BMI then reached baseline BMI (22.82 kg/m^2^, 95% CI (22.04–23.61)) after 3 years. No changes were noted in patients treated with NET. Significantly increased TC in patients after NCT (from 181.44 mg/dl, 95% CI (172.96–189.91 at baseline to 215.23 mg/dl, 95% CI (206.75–223.70); *p* < 0.05) followed by decrease to 176.15 mg/dL (95% CI (167.58–184.72)) after 3 years. No significant changes induced by NET. Fasting glucose increased from 95.36 mg/dL (95% CI (92.55–98.26)) at baseline to 111.36 mg/dL (92% CI (106.02–116.98) after NCT followed by decreased to 99.02 mg/dL (95% CI (95.71–102.45) after 3 years. No significant changes noted in the NET group. Significant increase in the NLR was noted in in NCT group (1.83, 95% CI (1.65–2.02) to 3.18, 95% CI (2.68–3.78); *p* < 0.01) followed by a decrease (1.42, 95% CI (1.04–1.93)) after three years and significant decrease in the NET group (1.98, 95% CI (1.78–2.21) to 1.43, 95% CI (1.20–1.72); *p* < 0.05) followed by an increase to 1.61 (95% CU (1.16–2.22)) after 3 years.	No association with cancer therapy–induced cardiotoxicity was investigated	Ryu et al., 2021 [289]
Arginine–nitric oxide metabolites	ECHO in n = 139 n = 32 experienced cardiotoxicity (23%)	DOX, cyclophosphamide followed by paclitaxel or DOX and cyclophosphamide followed by paclitaxel and trastuzumab	Blood samples at baseline, after cycle 2 of DOX (month 1), and after DOX completion (month 2)	Decreased levels of arginine and citrulline levels (*p* < 0.001) and increased ADMA levels (*p* < 0.001) at month 1 and persistent at month. Decrease in arginine and increase in ADMA and MMA were associated with cardiotoxicity at month 1 and a month 2, respectively. Increased levels of ADMA and MMA by 1.5-fold was associated with cardiotoxicity in response to DOX at month 2 (n = 117) with HR of 3.33 (95% CI (1.12, 9.96); *p* = 0.03)) and 2.70 (95% CI (1.35, 5.41); *p* = 0.005, respectively). No statistically significant association was observed between the levels of symmetric dimethylarginine (SDMA) (*p* = 0.90 month 1; *p* = 0.88 month 2), citrulline (*p* = 0.53 month 1; *p* = 0.28 month 2) and ornithine (*p* = 10 month 1; *p* = 0.06 month 2) at either time point.	Reduction in LVEF by ≥10% from baseline to LVEF < 50%	Finkelman et al., 2017 [291]
Lipoprotein subfractions and circulating metabolites	n = 250	- Six anthracycline-based courses (5-FU, epirubicin and cyclophosphamide) and/or - Four anthracycline-based courses followed by docetaxel and/or - Patients with hormone responsive tumors were treated with either tamoxifen or aromatase inhibitors and/or - Patients with HER2-positive breast cancer received trastuzumab plus docetaxel - Treatment groups included: - Group 1 (n = 47): Surgery, Radiotherapy, chemotherapy, endocrine treatment - Group 2 (n = 22): Surgery, Radiotherapy, chemotherapy - Group 3 (n = 35): Surgery, Radiotherapy, Chemotherapy with or without endocrine treatment and trastuzumab - Group 4 (n = 68): Surgery, Radiotherapy, Endocrine treatment - Group 5 (n = 78): surgery, radiotherapy	Serum samples were collected prior to RT (T1, n = 229), after RT completion (T2, n = 211), at 3 months (T3, n = 198), 6 months (T4, n = 195) and 12 months (T5, n = 146) Long-term survival and disease recurrence were assessed 10 years after enrolment to the study	Changes in lipoprotein composition (increased levels of LDL-cholesterol, lower levels of HDL-cholesterol, apo-A1 and apo-A2) was noted after treatment. VLDL-4 triglyceride levels were significantly decreased in patients treated with chemotherapy with or without endocrine treatment. All treated patients showed significantly lower levels in VLDL-5 triglyceride levels. Lysine (group 1: 0.018; group 2: 0.062; group 3: 0.036; group 4: 0.041), glutamate (group 1:0.081; group 2: 0.199; group 3: 0.251; group 4: −0.258) and formate (group 1: −0.322; group 2: −0.091; group 3: 0.167; group 4: 0.217) levels were upregulated during treatment whilst lower levels of glutamine (group 1: -0.091; group 2; −0.167; group 3; −0.126; group 4: 0.090) and lactate (group 1: −0.040; group 2: 0.124; group 3: −0.147; group 4: 0.174) were observed (linear mixed model analysis of longitudinal changes).	/	Giskeødegård et al., 2022 [290]
71 metabolites, previously associated with CV diseases	n = 19 cardiotoxicity n = 19 no cardiotoxicity	DOX, cyclophosphamide, paclitaxel and trastuzumab	ECHO at baseline and every 3 months Blood samples collected at baseline, 3 months (after completion of DOX) and 6 months (after completion of paclitaxel and trastuzumab)	Metabolites related to citric acid cycle, purine and pyrimidine were significantly altered (*p* < 0.05) in patients with cardiotoxicity. Patients without cardiotoxicity: - Showed increased levels of citric acid at 3 months (median change 10.2%, *p* = 0.04) and at 6 months (median change 12.9%, *p* = 0.04) compared to baseline. - Increased aconitic acid levels at 3 months (median change 24.6%, *p* = 0.05) and at 6 months (median change 25.2%, *p* = 0.009) compared to baseline. Patient with cardiotoxicity: - Showed stable or minor decrease citric acid levels at 3 months (median change −3.2%, *p* = 0.14) and at 6 months (median change -6.5%, *p* = 0.02). - No significant change in aconitic acid compared at 3 and 6 months compared to baseline. - Increased levels of purine metabolites: inosine (*p* = 0.002), hypoxanthine (*p* = 0.004), uric acid (*p* = 0.6) at 3 months compared to baseline and pyrimidine metabolites: pseudouridine (*p* = 0.05) and orotic acid (*p* = 0.003) at 3 months compared to baseline. No statistically significant differences noted in the levels of glucose/fructose/galactose (monosaccharides) or pyruvic acid in patients with or without cardiotoxicity.	LVEF decline by >10% resulting to LVEF < 55% compared to baseline	Asnani et al., 2020 [292]
Methylation signature of PBMCs using infinium HumanMethylation450 BeadChip	n = 9 abnormal LVEF n = 10 normal LVEF	DOX and cyclophosphamide	Blood samples were collected at baseline and after the first cycle of chemotherapy	A total of 14,883 and 18,718 differentially methylated CpGs were identified prior to and after the first cycle of chemotherapy, respectively, and correlated with changes in LVEF (*p* ≤ 0.05). Significant differential methylation was noted in *SLFN12*, IRF6 and *RNF39*. *SLFN12* and *IRF6* were hypermethylated and transcriptionally downregulated after the first cycle of chemotherapy compared to the baseline in all treatment groups (*IRF6 p* = 0.8837 normal LVEF; *IRF6 p* = 0.8119 abnormal LVEF; SLNF12 *p* = 0.5718 normal LVEF; SLNF12 *p* = 0.8575 abnormal LVEF).	Decrease in LVEF by >10% or LVEF < 50% compared to baseline	Bauer et al., 2021 [293]
High-throughput proteomic profiling using plasma samples	Case/control pairs 1 and 2 Case/control pair 3 Validation study of 35 participants	DOX plus cyclophosphamide followed by trastuzumab and paclitaxel.	ECHO at baseline, after DOX treatment completion and every 3 months of trastuzumab treatment. Blood samples collected at baseline, during treatment, upon diagnosis of CTRCD and, after CTRCD diagnosis.	862 proteins were identified using liquid chromatography-mass spectrometry (LC-MS) from case/control pairs 1 and 2 and 1360 proteins from case/control pair 3. IgE was significantly upregulated (5- to 58-fold; *p* = 0.018) in the control groups (non-cardiotoxicity patients) compared to the patients with cardiotoxicity at baseline and at all time points. This was verified (n = 35) using Luminex assay. Baseline IgE was higher in the non-cardiotoxicity group (mean 498.8 ng/mL ± 401.0; median 389.3 ng/mL with range 60.5–1392.1) compared to the cardiotoxicity group (mean 234.9 ng/mL ± 285.9; median 167 ng/mL with range 23.2–1059.2). In addition to IgE, baseline IgG4 levels and IgE related cytokines such as IL4, IL5 and IL17 were all lower in the case group compared to the control group.	Cardiotoxicity: LVEF reduction by ≥10% resulting in LVEF < 50% from baseline plus symptoms of HF Normal: LVEF change by <10% and LVEF > 50%	Beer et al., 2016 [294]
MMP2, MMP9	Month 12 n = 114 had anthracycline induced cardiotoxicity (AIC) of NYHA class I-III n = 70 no AIC After BC remission: Group 1: n = 54 (with AIC) Group 2: n = 60 (without AIC)	DOX and cyclophosphamide or DOX, and cyclophosphamide and docetaxel	Baseline, 12 months, and 24 months of treatment	Group 1: Elevated levels of MMP2: by 8% (*p* = 0.017) from 376.8 (329.5; 426.7) to 481.4 (389.8; 518.7) pg/mL and MMP9: by 18.4% (*p* < 0.001) from 23.6 (21.4; 24.6) to 26.0 (23.3; 27.0) pg/mL at 24 months. Group 2: MMP2 and MMP9 levels were lower at month 24. Increased levels of MMP2 and MMP9 could predict anthracycline induced CHF (AUC = 0.64; *p* = 0.013 and AUC = 0.9; *p* < 0.001, respectively). The presence of gene polymorphisms of MMP2 (rs243865) and MMP9 (rs3918242) were associated with anthracycline induced adverse events (OR = 4.76; *p* = 0.029; OR = 15.23; *p* < 0.000, respectively).	Decreased LVEF > 10% or LVEF < 55% with HF and NT-proBNP > 125pg/mL at 12 months after chemotherapy completion	Grakova et al., 2022 [284]
GDF-15	n = 30 placebo n = 33 perindopril n = 31 bisoprolol	DOX, carboplatin, trastuzumab (TCH) or 5-FU, epirubicin, cyclophosphamide (FEC) followed by docetaxel and trastuzumab	Baseline, post-cycle 4, post-cycle 17	Increase in GDF-15 at post-cycle 4 (+130 ± 150%, *p* ≤ 0.001) compared to baseline.	LVEF and GLS changes	Kirkham et al., 2022 [285]
CRP	n = 121	Epirubicin (with or without metoprolol succinate or candesartan) in combination with 5-FU and cyclophosphamide	Baseline and after completion of treatment	Increased levels of CRP after epirubicin treatment (*p* = 0.002) compared to baseline.	Cardiotoxicity was defined as previously described [67]	Gulati et al., 2017 [92]
hs-CRP	n = 56 (HER2-negative BC patients)	Adjuvant adriamycin, cyclophosphamide, or adriamycin, cyclophosphamide, taxane	Before each cycle, 12, 24 and 48 weeks after treatment completion	Elevated levels of hsCRP in response to chemotherapy (median levels 8.4 (4.95–16.5).	Changes in LVEF (not specified)	Hasan et al., 2021 [295]
hs-CRP GDF-15	n = 78 (HER2-positive BC patients)	DOX, cyclophosphamide followed by paclitaxel and trastuzumab	Baseline, every 3 months until month 15	Increased levels of hs-CRP and GDF-15 in response to therapy from baseline to visit 2 (mean changes between baseline and visit 2 (3.5 ± 5.0 mg/l, *p* < 0.001 and 575.4 ± 291.5 ng/l *p* < 0.001, respectively).	Cardiotoxicity was defined as previously described [67]	Ky et al., 2014 [105]

Lower levels of arginine after administration of DOX were associated with a significantly higher risk of DOX-induced cardiotoxicity in breast cancer patients (n = 124) at the early stages of treatment (HR = 0.78, 95% CI (0.64, 0.97); *p* = 0.02). Future studies are required to assess the relationship between the NO metabolites with other echocardiographic parameters, other than LVEF.

Asnani et al., 2020 [292], in a prospective study, revealed that HER2-positive breast cancer patients, who experienced CTRCD presented with statistically significantly altered levels of metabolites related to citric acid cycle, purine, and pyrimidine metabolites compared to patients without cardiotoxicity. In particular, citric acid levels were lower in the cardiotoxicity group compared to the baseline and to the non-cardiotoxicity group at 3 months (*p* = 0.008) and 6 months (*p* = 0.007) of treatment. Decrease in citric acid levels significantly correlated with changes in LVEF (R = 0.55, *p* = 0.01) The authors acknowledged that metabolic changes may also be attributed to tumor lysis and/or nephrotoxicity induced by DOX, even though all patients had normal renal function at baseline as indicated by normal plasma creatinine levels. Additional studies with larger sample sizes are required to further evaluate the role of metabolic changes in the development of anthracycline-induced cardiotoxicity.

Further investigations in cancer therapy-induced metabolic modulation in breast cancer patients, revealed that trastuzumab treatment impaired mitochondrial activity, and affected cardiac energy metabolism, calcium metabolism, and contractile capacity of human iPSC-CMs [296].

#### 3.4.4. Peripheral Blood Mononuclear Cells (PMBCs)

Bauer et al., 2021 [293], demonstrated that the methylation status of peripheral blood mononuclear cells (PBMCs) could predict the LVEF reduction after treatment with DOX in HER2-negative breast cancer patients. Specifically, a total of 14,883 differentially methylated CpGs were identified at baseline and were associated with a reduction in LVEF in response to DOX treatment. Three genes, the *Schlafen Family Member 12* (*SLFN12)*, *Interferon Regulatory Factor* 6 (*IRF6),* and the *Ring Finger Protein 39* (*RNF39)* were found to possess statistically significant differentially methylated regions (DMRs) in patients with cardiotoxicity. This study led to preliminary results based on a small sample size resulting in data variation and weaker correlation with CTRCD further indicating the need for larger cohort studies for evaluating the role of the methylation signature of PBMCs for the prediction of DOX-induced cardiotoxicity.

#### 3.4.5. Immunoglobulin E (IgE)

High-throughput proteomics analysis revealed three proteins with the largest differences in patients with cardiotoxicity (case) compared to patients without cardiotoxicity (control), which comprised: Immunoglobulin E (IgE), with significantly lower levels in patients with cardiotoxicity; the dopamine beta-hydroxylase (DBH) and cathepsin S (CTSS), both at higher levels in patients with cardiotoxicity compared to the control group [294]. The validation study focused on the levels of IgE since IgE showed the most significant differences (from 5- to 58-fold) in cardiotoxicity patients. Of note, the IgE levels in cancer patients with cardiotoxicity were similar to healthy volunteers, suggesting that IgE is elevated via an unknown mechanism. Overall, elevated levels of IgE decreased the risk of cardiac dysfunction indicating a cardioprotective effect of IgE in patients treated with DOX and trastuzumab. In this study, the two remaining biomarkers identified, DBH and CTSS, were not further evaluated due to a lack of high-throughput validation assays.

#### 3.4.6. C-Reactive Protein (CRP)

C-reactive protein (CRP), an inflammatory marker, has been assessed as a biomarker for the detection of CTRCD. Previous studies were identified in this review to show higher or lower levels of CRP in response to chemotherapy in breast cancer patients [92,163,286,295] with no clear association with the onset of CTRCD. Other studies found no association between the levels of CRP and subsequent cardiotoxicity [105].

#### 3.4.7. Growth Differentiation Factor 15 (GDF-15)

Growth differentiation factor 15 (GDF-15) is a hormonal peptide of the transforming growth factor-β superfamily. Increased levels of GDF-15 were noted in cardiomyocytes during ischemia–reperfusion injury and myocardial infarction, which led to the notion that GDF-15 can be used as a marker of cardiac failure [297]. Upregulated levels of GDF-15 (*p* < 0.05) were observed in response to treatment with DOX and trastuzumab in breast cancer patients, however, no statistically significant association was noted with CTRCD. A study by Tromp et al., 2020 [153], showed a strong association of GDF-15 (*p* = 0.002) with changes in LVEF in late breast cancer survivors after correction for age, BMI, existing or past CV disease, treatment with radiotherapy. In addition, Ky et al., 2014 [105], showed significantly elevated levels of GDF-15 in response to the early stages of chemotherapy in breast cancer patients. Kirkham et al., 2022 [285] showed a statistically significant increase in GDF-15 levels at post-cycle 4 of trastuzumab compared to baseline in HER2-positive breast cancer patients (*p* ≤ 0.001). All studies support that further studies with a larger number of patients are required to evaluate the predictive role of GDF-15 in CTRCD and overcome inconsistencies.

#### 3.4.8. Other Biomarkers under Investigation

In addition to above-mentioned biomarkers, other potentially promising biomarkers are currently under investigation for their utility in the detection and/or prediction of CTRCD including: coronary artery calcification [298], endothelin-1 [299], neuregulin-1 [285,300], plasma bioactive adrenomedullin (ADM) [301], fragmented QRS (fQRS) [302], D-dimer [303], soluble fms-like tyrosine kinase receptor (sFlt)-1 [105], soluble ST2 (sST2) [145,149,171,279,304], CK-MB [134,286], *topoisomerase II α* gene (*TOP2A*) [305], cardiac myosin light chain 1 (cMLC-1) [306], vascular endothelial growth factor (VEGF) [93], placental growth factor (PIGF) [93,98], procollagen-derived type-I C-terminal-propeptide (PICP) [307], epicardial adipose tissue (EAT) volume [308,309], circulating bilirubin [310], hemopexin [311], glycated hemoglobin (HbA(1)c) [103], advanced oxidation protein products (AOPP) [102], human resistin [312] and vascular adhesion molecule 1 (VCAM-1) [117].

With respect to the genetic factors, the following are currently under investigation with potentially promising outcomes: polymorphisms in *p53* (rs1042522) [313,314], *NOS3* (rs1799983), *NADPH* oxidase (rs4673), *GPX1* (rs1050450) [313], *RARG* [37,315], *POLRMT* [316] *DPYD* [317] and *ABCC1* [318]. Interestingly, Varkoly et al., 2022 [319], demonstrated for the first time that circulating RNA virus gene sequences (e.g., influenza orthomyxovirus) were detected in the blood samples of patients with hematological malignancies or breast cancer and were associated with myocarditis and lower LVEF in response to chemotherapy.

## 4. Discussion

CTRCD poses a critical problem in the immediate and long-term management of breast cancer patients. As an anti-cancer therapy-related complication, myocardial damage and HF are associated with high morbidity and mortality, requiring often lifelong, specialist cardiological treatment. Anthracycline-based chemotherapy and some types of targeted therapy have been mostly associated with CV toxicity in breast cancer [11,16,99,320]. Acknowledging the extent but also the potentially severe consequences of CTRCD, the European Society of Cardiology introduced clinical practice guidelines for its assessment and management [1,7,13,18,19,67]. However, in the era of precision medicine, what remains a critical challenge is to identify reliable biomarkers for the diagnosis, the follow-up, and perhaps most importantly, for the prediction of cardiotoxicity in breast cancer patients receiving systemic therapy. In this systematic review, we sought to improve current insights on the use of existing but also potentially promising novel biomarkers as indicators and/or predictors of CTRCD in this population.

Through this review, it is confirmed that traditional biomarkers, namely troponins, the natriuretic peptides, and the LVEF are the most frequently used CTRCD biomarkers in clinical trials/ studies, and in clinical practice, albeit not universally. Their strengths and limitations are documented. Critical reappraisal of the latest international guidelines [2,321,322] performed by Clerico et al., 2023 [82] highlights the importance of baseline and subsequent serial measurement of NPs and cTn for each patient subjected to cardiotoxic cancer therapy. Cardiac troponins and natriuretic peptides can be indicators of cardiomyocyte injury, but their predictive capacity for the onset of cardiotoxicity lacks reliability [17]. Similarly, LVEF can be used as a strong indicator of cardiac dysfunction, but it lacks sensitivity for early detection of subclinical cardiac impairment, which may be crucial for the management of certain subgroups of breast cancer patients, such as the elderly [8,18,19,22,24]. Further studies are required to define the exact unique or complementary roles of troponins, BNPs, and the LVEF as detection, monitoring, and/or predictive biomarkers of CTRCD in different breast cancer subtypes, different therapy regimens, and in association with different patient characteristics. Despite the potential of the currently available cardiac-specific markers in CTRCD, the predictive capacity of such markers remains elusive. For this reason, clinical studies explored the role of emerging/novel biomarkers as predictors and/or indicators of the early stages of CTRCD and these are extensively discussed in this review.

Several promising emerging markers are reported in the literature including SNPs, microRNAs, MPO, Gal-3, and MMPs. Four out of six SNP studies identified through the current review, showed a strong association of the SNP *HER2* lle655Val with trastuzumab-induced cardiotoxicity in patients with breast cancer [211,214,215,216]. Additional studies have validated the role of *HER2* codon 655 A>G in increasing the risk of trastuzumab-induced cardiotoxicity in French, Chinese, and Canadian patients [221,229]. In contrast, two studies have demonstrated no association between *HER2* lle655Val and CTRCD suggesting that additional studies are required to clarify the role of this SNP in the prediction and detection of CTRCD in breast cancer patients [212,213]. Based on these conflicting results, studies have focused on risk factors that may influence trastuzumab-mediated cardiotoxicity in the presence of the *HER2* lle655Val SNP including alcohol consumption, BMI, diabetes, hypertension, and combination treatment with anthracyclines [229]. Importantly, it is supported that allele frequencies of the HER2 SNPs differ based on ethnicity and so the mechanism through which trastuzumab exhibits its cardiotoxicity may differ among different races [221,223]. In addition, five SNPs (rs9316695, rs28415722, rs7406710, rs11932853, and rs8032978) have been identified to predict trastuzumab-induced cardiotoxicity at baseline whilst two of them rs8032978 and rs7406710 were the ones with the strongest association [223,237]. The rs8032978 and rs7406710, which showed the strongest association with trastuzumab-induced cardiotoxicity, are located 44 kb downstream of the *proprotein convertase subtilisin/kexin type 6* (*PCSK6*) and 1.5 kb downstream of the *fascin actin-bundling protein 2, retinal* (*FSCN2*), respectively. Dysregulation of PCSK6 and FSCN2 expression and/or function has been associated with poor cardiac recovery and impaired cardiac contraction, respectively [223,323,324].

Several studies have investigated the promising role of miRNAs as clinical biomarkers for the prediction of CTRCD in breast cancer patients. Indeed, a total of 445 miRNAs were differentially expressed in response to CTRCD in breast cancer patients with a statistically significant correlation with LV dysfunction. Previous studies have identified miR-3135b-5p to be related to obstructive coronary artery disease and implicated in pathological processes associated with CHF including cardiac muscle contraction and calcium ion transport [325,326]. Significant upregulation of miR-3135b-5p was noted in patients, who experienced CTRCD, indicating its promising role in the diagnosis of CTRCD [252]. Larger patient cohorts are needed to validate the miRNA biomarker panel identified as diagnostic tools for chemotherapy-induced cardiotoxicity.

Evidence also indicated that assessing markers such as MPO may enhance the prediction capacity of doxorubicin and trastuzumab-induced cardiac dysfunction. Several studies support the notion that both serum biomarkers of cardiac injury and cardiac imaging should be considered for evaluating cardiac function in patients with a high risk of developing CTRCD. However, conventional cardiac imaging enables the detection of late-onset cardiac dysfunction, and hence newly established circulating biomarkers such as MPO can be used as promising biomarkers for the detection of the early onset CTRCD even before detected by echocardiographic assessment. MPO may be utilized for accurate and consistent detection of acute, subclinical cardiac dysfunction prior to irreversible CHF [286].

Based on the current review, the role of Gal-3 in the monitoring of CTRCD remains unclear and the data generated lead to controversial results [271,277,327]. Inconsistencies among studies may be attributed to the heterogeneity of the patient population between studies including the timeline assessed, the sensitivity of biomarker assays used, cancer type/stage, and doses of chemotherapy. Comparing Gal-3 and NT-proBNP, it seems that the role of the latest as a predictor/indicator of CTRCD is well-investigated and understood. Based on the studies presented in this review, it seems that NT-proBNP seems to be superior to Gal-3 in predicting cardiotoxicity [123,124,136]. Similarly, MMPs comprise another potential biomarker that could be used to assess the risk of CTRCD in breast cancer patients particularly on the late onset of anthracycline-induced cardiotoxicity [284,285], but further studies are needed to better evaluate the predictive role of MMP2, MMP9 and other MMPs for early and late stages of cardiotoxicity in breast cancer patients.

Other markers have emerged in recent years as possible biomarkers of prediction of CTRCD. In this review, we have presented currently available results on a number of such potentials. Pilot studies have revealed distinct biomarker profiles with significant differences in chemokine/cytokine abundance and/or immune signatures in patient groups with cardiotoxicity in response to cancer therapy. CCL23 were amongst the biomarkers assessed that demonstrated a persistent increase in the cardiotoxicity group at all time points (baseline, after the first and second cycle of Dox, respectively), tested compared to the non-cardiotoxicity group and therefore its utility as a biomarker of cardiotoxicity needs to be further elucidated [287]. In another example, Yu et al., 2018 [287], highlighted that CXCL1, which was significantly lower in patients with cardiotoxicity, was previously shown to possess cardioprotective effects in cardiomyocytes of mouse neonatal exposed to DOX treatment [328], suggesting that alterations in immune response proteins and inflammatory-associated proteins may induce susceptibility to DOX-induced cardiotoxicity and may be useful predictors of DOX-mediated cardiotoxic effects.

Another potential surrogate marker, with increasing popularity, is the measurement of elevated NLR values in breast cancer patients during anthracycline and/or trastuzumab-based therapies [288]. The cut-off value of NLR indicating LV dysfunction was set to >2.58 by Baruch et al., 2022 [288], which is in concordance with previous studies using a median NLR cut-off value of 3 (13 studies) to predict overall survival and at 2.5 (10 studies) to predict disease-free survival in breast cancer patients [329]. Patients with NLR ≥ 2.58 had a twice as high risk for LV GLS reduction by ≥10% (50% versus 20%, *p* = 0.009) in response to anthracycline-based chemotherapy, although, it is acknowledged that high NLR value is not the only independent predictor, since trastuzumab treatment could also predict LV GLS reduction models [288].

In addition to the cardiotoxic potential, multimodal therapeutic regimens for breast cancer have been associated with alterations in the metabolic profiles in patients. Ryu et al., 2021 [289], demonstrated that neoadjuvant chemotherapy induced a significant increase in total cholesterol and fasting glucose compared to baseline. In addition to the distinct chemokine and immunological profiles identified in patients who experienced CTRCD, studies revealed that patients develop different atherogenic lipid profiles, depending on the therapeutic regimens received [290]. Interestingly, non-survivors showed to have different lipoprotein profiles compared to patients who survived 10 years after enrolment in the study [290]. As recently highlighted by Guha et al., 2022 [330], extreme attention to the lipoprotein profiles of patients and the dyslipidemic potential of certain chemotherapeutic agents is needed during treatment and/or follow-up in order to allow targeted interventions for reducing the risk of CV diseases and preventing cardiotoxicity and adverse events. In particular, elevated levels of triglycerides are a frequent complication associated with increased overall mortality in patients with breast cancer [331].

Patients with cardiotoxicity demonstrated significantly decreased levels of citric acid metabolites with minor changes in aconitic acid and a significant increase in purine and pyrimidine metabolites compared to the baseline and to patients without cardiotoxicity in response to anthracycline-based chemotherapy and trastuzumab. Of note, reduced levels of citric acid metabolites occurred at the early stages of treatment with anthracyclines plus cyclophosphamide prior to administration of trastuzumab [292]. These findings suggest that assessing changes in nucleoside metabolism and citric acid metabolites may assist in the development of early risk stratification strategies and the use of cardioprotective compounds in patients with a high risk of anthracycline cardiotoxicity. However, further studies are warranted to further explore the role of circulating metabolites in the context of CTRCD.

Findings by Finkelman et al., 2017 [291] showed an association of arginine–nitric oxide (NO) metabolites and dox-induced cardiotoxicity in breast cancer patients, with elevated levels of ADMA and decreased levels of arginine. A more recent study by Giri et al., 2019 [332], denoted a statistically significant increase in the serum levels of nitrite (*p* < 0.001) and the endothelial marker, von Willebrand factor (vWF) (*p* < 0.001), in cancer patients treated with chemotherapy compared to cancer patients received no chemotherapy. The elevated levels of nitrite and vWF were in parallel with higher levels of cTnI and cTnT (even though not statistically significant, *p* > 0.05), making both vWF possible diagnostic indicators of subclinical cardiac dysfunction.

Proteomics analysis revealed that baseline IgE in cancer patients who experienced cardiotoxicity in response to DOX and trastuzumab was significantly lower compared to cancer patients that did not experience cardiotoxicity. Monitoring the levels of IgE prior to treatment may indicate patients that are most likely to be at higher risk to develop cardiac dysfunction. However, further studies are needed to demonstrate whether IgE and/or other Ig subtypes may possess the benefit to predict the onset of anthracycline and trastuzumab-induced cardiac toxicity [294].

In addition to the circulating biomarkers for the prediction and/or detection of CTRCD, cardiac imaging modalities are currently under investigation for the early detection and/or prediction of CTRCD. Although extensive discussion of all modalities of cardiac imaging is beyond the scope of this review, the current guidelines recommend the use of transthoracic echocardiography (TTE) (2D- or 3D-TTE, CMR) for the detection of CTRCD [2]. Emerging imaging techniques are currently investigated including the 2D- and 3D-STI, RT-3DE, and ^123^I-mIBG scintigraphy [146,151,155,156,157,162,164,168].

Through this review, we have identified several studies on the prospective long-term follow-up of patients with breast cancer subjected to treatment with cardiotoxic therapies [98,136,149,153,290]. Several randomized multi-center prospective studies (i.e., PROACT (NC NCT03265574)) [333] or longitudinal studies (i.e., CardioToX (NCT04790266)) [334], are currently ongoing aiming to assess the use of cardiac imaging and circulating biomarkers in the diagnosis of CTRCD in breast cancer patients treated with cardiotoxic cancer therapy including anthracyclines and/or trastuzumab.

In addition, several studies in this review included metastatic breast cancer patients amongst their target population and investigated the potential of emerging biomarkers in the detection of CTRCD in these patients [128,130,183,222,255,298,304,329]. Noticeably, some of these studies, have highlighted the differential expression of specific biomarkers (e.g., sFas, sFasL, NT-proBNP) in metastatic breast cancer patients compared to non-metastatic [104,128].

Several limitations have been acknowledged in the studies or trials included in this review. An important issue concerning all clinical studies or trials is the definition of CTRCD used by the authors, which evidently varies across the scientific literature, making comparisons between different studies, challenging. The variability of results observed in studies concerning different biomarkers might be attributed to the different therapeutic regimens, the sample size of the studies/ trials, the time points at which the biomarkers are assessed, and/or the different assays used to detect each marker. In addition, a lack of reproducibility was evident for certain biomarkers across clinical studies. This was noted in the case of the SNP *HER2* lle655Val for which even though the majority of the prospective clinical studies demonstrated a strong association with CTRCD, still this association was not reproducible in other clinical studies. In addition, evidence suggests Gal-3 as a potential indicator of CTRCD, still several studies demonstrated a lack of association. In addition, it should be acknowledged that radiotherapy, a widely applicable therapeutic modality in breast cancer alongside systemic therapy, may trigger the development of CV diseases. Even though evidence suggests that radiotherapy can contribute to CTRCD and should be further investigated as a contributing factor, the impact of radiotherapy in the development of CTRCD was beyond the scope of this review.

## 5. Conclusions

We have conducted an extensive literature review to further increase the existing knowledge and share awareness on the current and/or newly investigated biomarkers for the detection and/or prediction of CTRCD in patients with breast cancer. The strengths and weaknesses of the current state-of-the-art in the management of CTRCD in breast cancer patients using biomarkers have been highlighted, aiming to pave the way for the development of a more advanced and accurate risk stratification model for the prediction and/or early detection of CTRCD in this target population. We have discussed the available evidence on three “classical biomarkers” (troponins, NPs, and LVEF/strain changes), five “emerging biomarkers” (SNPs, miRNAs, MPO, Gal-3, and MMPs), several “other biomarkers” (inflammatory, NLR, metabolism, PBMCs, IgE, CRP, and GDF-15), and provided a list of numerous newly investigated biomarkers in the field of precision medicine for the assessment of CTRCD in breast cancer patients. The review has identified biomarker tools with promising predictive capacities for the early stages of CTRCD even before changes are detected by the traditional biomarkers. This review further highlights the need for larger additional and prospective clinical studies with sufficient statistical power for the validation of existing and/or newly discovered indicators and/or predictors of CTRCD. Overall, a combination of both serum and imaging biomarkers is encouraged for the best and most precise risk stratification of patients at risk to develop cardiotoxicity in response to breast cancer therapy. Towards this direction, the co-authors of this study aim to explore the utility of novel biomarkers and develop a risk stratification model for the prediction and/or early detection of CTRCD in breast cancer patients through the CardioCare project funded by Horizon 2020 (945175) [335].

## Data Availability

Data that support the findings of this study can be provided by the corresponding authors upon request.

## Supplementary Materials

The following supporting information can be downloaded at: https://www.mdpi.com/article/10.3390/cancers15133290/s1, Table S1: Studies identified for the traditional biomarkers; Table S2: Studies identified for the emerging biomarkers.
