# Supplementary material for: New Insights in the Era of Clinical Biomarkers as Potential Predictors of Systemic Therapy-Induced Cardiotoxicity in Women with Breast Cancer: A Systematic Review
Source: Cancers (Basel). 2023 Jun 22;15(13):3290. doi: 10.3390/cancers15133290 (PMC10340234; doi:10.3390/cancers15133290)
Supplement: Supplementary file 1 [file cancers-15-03290-s001.zip › cancers-2416704-supplementary.pdf]

Table S1. Studies identified for the traditional biomarkers.

	References		References
**Troponins**	Shafi *et al.*, 2017 [84]<br>Zardavas *et al.*, 2017 [10]<br>Blaes *et al.*, 2015 [85]<br>Díaz-Antón *et al.*, 2022 [86]<br>Howden *et al.*, 2021 [87]<br>Pillai *et al.*, 2022 [89]<br>Bisoc *et al.*, 2020 [90]<br>Goel *et al.*, 2019 [91]<br>Gulati *et al.*, 2017 [92]<br>Gullo *et al.*, 2019 [93]<br>Kirkham *et al.*, 2021 [94]<br>Lakhani *et al.*, 2021 [95]<br>Malik *et al.*, 2016 [96]<br>Ponde *et al.*, 2018 [97]<br>Putt *et al.*, 2015 [98]<br>Rüger *et al.*, 2020 [99]<br>De Sanctis *et al.*, 2021 [100]<br>De Vries Schultink *et al.*, 2018 [101]<br>Silva *et al.*, 2017 [102]<br>Simões *et al.*, 2021 [103]<br>Ürun *et al.*, 2015 [104]<br>Ky *et al.*, 2014 [105]<br>Anber *et al.*, 2019 [106]<br>Dhesy-Thind *et al.*, 2019 [107]<br>Dovganych *et al.*, 2022 [108]<br>Finke *et al.*, 2021 [109]<br>Florido *et al.*, 2019 [110]<br>Henriksen *et al.*, 2022 [111]<br>Hidayat *et al.*, 2020 [112]<br>Hinrichs *et al.*, 2020 [113]<br>Isemede *et al.*, 2022 [114]<br>Jones *et al.*, 2017 [115]<br>Mokuyasu *et al.*, 2015 [116]<br>Tzolos *et al.*, 2020 [83]<br>Rosenkaimer *et al.*, 2022 [117]<br>Posch *et al.*, 2022 [118]<br>Ben Kridis *et al.*, 2020 [119]	**NPs**	Andersson *et al.*, 2021 [122]<br>Blancas *et al.*, 2020 [123]<br>Bouwer *et al.*, 2019 [124]<br>Alves *et al.*, 2022 [125]<br>Matos *et al.*, 2016 [126]<br>Lu *et al.*, 2019 [127]<br>Bisoc *et al.*, 2020 [90]<br>Díaz-Antón *et al.*, 2022 [86]<br>Ky *et al.*, 2014 [105]<br>De Iuliis *et al.*, 2015 [129]<br>Demissei *et al.*, 2020 [130]<br>Demissei *et al.*, 2019 [131]<br>Dong *et al.*, 2022 [132]<br>El-Sherbeny *et al.*, 2019 [133]<br>Goel *et al.*, 2019 [91]<br>Grela-Wojewoda *et al.*, 2022 [134]<br>Gulati *et al.*, 2017 [92]<br>Howden *et al.*, 2021 [87]<br>Liang *et al.*, 2016 [135]<br>Malik *et al.*, 2016 [96]<br>Patel *et al.*, 2021 [136]<br>Ponde *et al.*, 2018 [97]<br>Putt *et al.*, 2015 [98]<br>Rüger *et al.*, 2020 [99]<br>De Sanctis *et al.*, 2021 [100]<br>De Vries Schultink *et al.*, 2018 [101]<br>Simões *et al.*, 2021 [103]<br>Skovgaard *et al.*, 2014 [137]<br>Anber *et al.*, 2019 [106]<br>Caram *et al.*, 2015 [138]<br>Dores *et al.*, 2013 [139]<br>Finke *et al.*, 2021 [109]<br>Hinrichs *et al.*, 2020 [113]<br>Isemede *et al.*, 2022 [114]<br>Kittiwarawut *et al.*, 2013 [140]<br>Kouloubinis *et al.*, 2015 [128]<br>Rosenkaimer *et al.*, 2022 [117]<br>Ben Kridis *et al.*, 2020 [119]<br>Özbay *et al.*, 2022 [141]<br>Dhir *et al.*, 2019 [142]<br>Esmaeilzadeh *et al.*, 2022 [143]<br>Feng *et al.*, 2021 [144]<br>Posch *et al.*, 2022 [118]<br>Gherghe *et al.*, 2022 [145]

**LVEF**	**References**<br>Stoodley *et al.*, 2013 [146]<br>Stoodley *et al.*, 2013 [147]<br>Suerken *et al.*, 2015 [148]<br>Bulten *et al.*, 2015 [149]<br>Bulten *et al.*, 2014 [159]<br>Tahir *et al.*, 2022 [150]<br>Wang *et al.*, 2020 [151]<br>Tromp *et al.*, 2020 [153]<br>El-Sherbeny *et al.*, 2019 [133]<br>Goel *et al.*, 2019 [91]<br>Huang *et al.*, 2018 [171]<br>Huang *et al.*, 2022 [160]<br>Huang *et al.*, 2017 [161]<br>Rüger *et al.*, 2020 [99]<br>Skovgaard *et al.*, 2014 [137]<br>Todorova *et al.*, 2020 [163]<br>Lakhani *et al.*, 2021 [95]<br>Coutinho Cruz *et al.*, 2020 [164]<br>Dang *et al.*, 2016 [165]<br>Demissei *et al.*, 2019 [131]<br>Demissei *et al.*, 2020 [130]<br>Dong *et al.*, 2022 [132]<br>Dores *et al.*, 2013 [139]<br>Dovganych *et al.*, 2022 [108]<br>Ferreira *et al.*, 2022 [168]<br>Finke *et al.*, 2021 [109]<br>Gavila *et al.*, 2017 [169]<br>Hinrichs *et al.*, 2020 [113]<br>Honda *et al.*, 2017 [170]<br>Jordan *et al.*, 2014 [172]<br>Kibudde *et al.*, 2019 [173]<br>Kim *et al.*, 2019 [174]<br>Liu *et al.*, 2023 [175]<br>Lorenzini *et al.*, 2017 [176]<br>Mescher *et al.*, 2017 [177]<br>Shafi *et al.*, 2017 [84]<br>Sun *et al.*, 2016 [178]<br>Yamashita *et al.*, 2021 [179]<br>Abdallah *et al.*, 2020 [180]<br>Altaha *et al.*, 2020 [181]<br>Arciniegas Calle *et al.*, 2018 [182]<br>Ben Kridis *et al.*, 2020 [119]<br>Calvillo-Argüelles *et al.*, 2022 [183]<br>Chang *et al.*, 2020 [185]<br>Chang *et al.*, 2016 [184]<br>Chen *et al.*, 2019 [220]<br>Choe *et al.*, 2018 [186]<br>De Barros *et al.*, 2019 [187]<br>De Souza *et al.*, 2021 [188]<br>Demissei *et al.*, 2021 [166]

**LVEF**	Dhir *et al.*, 2019 [142]<br>Ferreira De Souza *et al.*, 2018 [167]<br>Florescu *et al.*, 2014 [158]<br>Gong *et al.*, 2019 [189]<br>Gripp *et al.*, 2018 [190]<br>Guan *et al.*, 2021 [191]<br>Guerra *et al.*, 2016 [192]<br>Hochstadt *et al.*, 2020 [193]<br>Hochstadt *et al.*, 2021 [194]<br>Jones *et al.*, 2022 [195]<br>Kirkham *et al.*, 2021 [94]<br>Monti *et al.*, 2020 [196]<br>Nakano *et al.*, 2016 [197]<br>Narayan *et al.*, 2017 [198]<br>Negishi *et al.*, 2013 [200]<br>Negishi *et al.*, 2018 [201]<br>Ong *et al.*, 2018 [202]<br>Özbay *et al.*, 2022 [141]<br>Park *et al.*, 2020 [203]<br>Perone *et al.*, 2022 [204]<br>Piotrowski *et al.*, 2013 [205]<br>Posch *et al.*, 2022 [118]<br>Reuvekamp *et al.*, 2016 [206]<br>Rushton *et al.*, 2017 [207]<br>Sato *et al.*, 2019 [208]<br>Seferina *et al.*, 2016 [209]<br>Tan *et al.*, 2022 [210]<br>Terui *et al.*, 2023 [211]<br>Tu *et al.*, 2022 [162]<br>Van der Linde *et al.*, 2022 [212]<br>Xu *et al.*, 2021 [213]<br>Yaylali *et al.*, 2016 [214]<br>Yersal *et al.*, 2018 [215]<br>Zhang *et al.*, 2021 [216]<br>Zhou *et al.*, 2020 [219]<br>Feng *et al.*, 2021 [218]<br>Gherghe *et al.*, 2022 [145]

Table S2. Studies identified for the emerging biomarkers.

**SNPs**	**References**<br>Tan *et al.*, 2020 [221]<br>Stanton *et al.*, 2015 [221]<br>Serie *et al.*, 2017 [226]<br>Roca *et al.*, 2013 [227]<br>Gómez Peña *et al.*, 2015 [228]<br>Lemieuz *et al.*, 2013 [229]<br>Schneider *et al.*, 2017 [231]<br>Gvaldin *et al.*, 2021 [233]<br>Li *et al.*, 2019 [234]<br>Li *et al.*, 2022 [235]<br>Sawyer *et al.*, 2016 [236]<br>Nakano *et al.*, 2019 [223]<br>Nyangwara *et al.*, 2023 [315]<br>Reinbolt *et al.*, 2015 [224]<br>Soria-Chacartegui *et al.*, 2021 [317]<br>Velasco-Ruiz *et al.*, 2021 [316]<br>Vivenza *et al.*, 2013 [225]<br>Vulsteke *et al.*, 2015 [318]<br>Shilov *et al.*, 2019 [314]<br>Udagawa *et al.*, 2022 [237]	**microRNAs**	**References**<br>Sánchez-Sánchez *et al.*, 2022 [251]<br>Lakhani *et al.*, 2021 [95]<br>Zare *et al.*, 2022 [252]<br>Feng *et al.*, 2021 [144]<br>Zhang *et al.*, 2020 [253]<br>Gioffré *et al.*, 2020 [254]<br>Gupta *et al.*, 2013 [255]<br>Holmgren *et al.*, 2016 [256]<br>Liu *et al.*, 2022 [257]<br>Qin *et al.*, 2018 [258]<br>Rigaud *et al.*, 2017 [259]<br>Todorova *et al.*, 2017 [260]<br>Yadi *et al.*, 2020 [261]<br>Zhu *et al.*, 2018 [262]<br>Pillai *et al.*, 2022 [89]<br>Frères *et al.*, 2018 [304]
**MPO**	**References**<br>Lakhani *et al.*, 2021 [95]<br>Wanderley *et al.*, 2022 [271]<br>Putt *et al.*, 2015 [98]<br>Ky *et al.*, 2014 [105]<br>Todorova *et al.*, 2020 [163]<br>Gullo *et al.*, 2019 [93]<br>Balmagambetova *et al.*, 2022 [303]<br>Demissei *et al.*, 2020 [130]	**Galectin-3**	**References**<br>Gulati *et al.*, 2017 [92]<br>Bulten *et al.*, 2015 [149]<br>Van Boxtel *et al.*, 2015 [279]<br>Ky *et al.*, 2014 [105]<br>Wanderley *et al.*, 2022 [271]<br>Patel *et al.*, 2021 [136]<br>Putt *et al.*, 2015 [98]<br>Balmagambetova *et al.*, 2022 [303]<br>Magalhaes *et al.*, 2015 [278]
		**MMPs**	**References**<br>Grakova *et al.*, 2022 [284]<br>Kirkham *et al.*, 2022 [285]
